# Virulence‐Selective Trap–Capture–Kill Antibacterial Nanostructures With Immune–Metabolic Regulation for Treating Implant‐Associated Infections

**DOI:** 10.1002/advs.202511045

**Published:** 2025-09-15

**Authors:** Xiaodong Hu, Jiaqi Zhong, Yujiong Chen, Botao Liu, Tianyu Du, Weilai Zhu, Minzhe Zheng, Hongze Liang, Jiahua Ni, Zhaoxiang Peng

**Affiliations:** ^1^ Affiliated Li Huili Hospital Ningbo University Ningbo 315040 China; ^2^ Health Science Center Ningbo University Ningbo 315211 China; ^3^ Peking University First Hospital Beijing 100034 China; ^4^ Key Laboratory of Advanced Mass Spectrometry and Molecular Analysis of Zhejiang Province School of Materials Science and Chemical Engineering Ningbo University Ningbo 315211 China; ^5^ College of Biological Science and Medical Engineering Donghua University Shanghai 201620 China

**Keywords:** antibacterial, bone metabolism, immune microenvironment, implant‐associated infections, TiO_2_ nanostructure

## Abstract

The treatment of orthopedic implant–associated infections (IAIs) presents significant challenges due to increasing drug resistance. This study reports a virulence‐selective trap–capture–kill antibacterial system for effectively treating persistent infections while promoting local bone tissue regeneration by regulating disordered immune and metabolic microenvironments caused by IAIs. Here, this work fabricates a nanostructured antibacterial system (F‐14‐TNA) based on a TiO_2_ nanotube array modified with a cationic pentacyclic coplanar backbone, fascaplysin derivative 14 (F‐14). The modified surface induces a unique virulence‐initiated chemotaxis behavior, with the TiO_2_ nanostructure selectively trapping hyperpathogenic bacterial strains from the infecting microbiota. These trapped bacterial strains are subsequently captured by the positively charged F‐14 surface via electrostatic force. The virulence‐selective bacteria are then killed via a reactive oxygen species–mediated pathway regulated by the phosphotransferase system cyclic adenosine monophosphate receptor protein cascade. In vitro and in vivo studies reveal that F‐14‐TNA has multiple biocompatibility functions, such as effective antibacterial activity, regulation of the immune microenvironment of bone infections, and recovery of disordered bone metabolism. The nanostructure developed in this study provides a novel approach for the treatment of IAIs.

## Graphical abstract

A virulence‐selective antibacterial system based on fascaplysin derivative 14‐modified TiO_2_ nanostructure (F‐14‐TNA) is developed for treating implant‐associated infections. F‐14‐TNA selectively traps hyperpathogenic bacteria via chemotaxis, captures them electrostatically, and kills them via reactive oxygen species‐mediated pathways. It concurrently regulates immune‐metabolic microenvironments, promoting M2 macrophage polarization and bone regeneration while inhibiting osteolysis, offering a potent dual‐functional strategy against resistant infections.

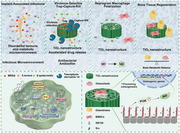

## 1 Introduction

Implant‐associated infections (IAIs) pose a major clinical challenges, involving the overuse of antibiotics in their treatment, thereby escalating the incidence of multidrug‐resistant bacteria and posing risks to public healthcare systems^[^ ^1^, ^2^ ^]^ Such challenges underscore the need for alternative therapeutic strategies.^[^ ^3^ ^]^ Recent advancements in the management of IAIs have emphasized on the development of antibiotic‐free strategies,^[^ ^4^, ^5^, ^6^ ^]^ including ions (Ga^2+^ and Ag^+^),^[^ ^7^, ^8^ ^]^ nanoenzymes,^[^ ^9^ ^]^ and photo/sono‐dynamic therapy.^[^ ^10^, ^11^ ^]^ However, these approaches failed to combine potent antibacterial and anti‐inflammatory efficacy with bone metabolic balance to facilitate tissue regeneration and biocompatibility. Strategies addressing both antibacterial efficacy and tissue regeneration have important clinical value and social significance with respect to resolving the increasing burden of IAIs in clinical settings, protection of patient health, and reduction of medical costs.

Traditional anti‐infection strategies for implants mainly focus on the design of antibacterial coatings for implant surfaces. However, in the case of drug‐eluting coatings and anchored antibacterial coatings, the diversity within the infecting microbiota is neglected. Notably, high‐virulence pathogens often migrate more rapidly to deep layers of the implant–tissue interface after biofilm formation and enter a metabolic dormant state. Traditional antibacterial coatings form a natural concentration gradient, causing difficulty in the eradication of deep, highly virulent antimicrobials. In this regard, exploiting chemotactic/physiotactic behavior of bacteria is a potential avenue for investigation. For instance, Xiao et al. showed that lysine‐loaded nanoparticles can stimulate bacterial chemotaxis, presenting a new method for modulating bacterial behavior for therapeutic applications.^[^ ^12^ ^]^ Similar to chemical gradient–based bacterial taxis, which has demonstrated therapeutic potential, physical gradients (e.g., nanotopography) have been reported to orchestrate bacterial migration patterns, offering distinct advantages for combating deep‐seated biofilm infections.^[^ ^11^, ^13^ ^]^ Unlike chemical cues that may degrade or diffuse unpredictably in biological environments, physical gradients provide spatiotemporally stable directional cues, enabling precise control over bacterial motility. High‐virulence bacteria of the same lineage often share common traits, such as high adhesiveness and overexpressed biodegradation enzymes. In this regard, TiO_2_ nanostructures (TNA), as a classic nanomechanical stimulus, markedly regulate the adhesion, proliferation, and osteogenic differentiation of cells and bacteria.^[^ ^14^ ^]^ Hence, we herein fabricated a unique TNA that controls surface chemical groups and exhibits a physical profile that initiates virulence‐selective bacterial chemotaxis. This can prove to be an extremely effective therapeutic strategy, as in this case, high‐virulence bacteria can initially be trapped on the surface of the TNA through chemotaxis and then eliminated.

Following the effective trapping of bacteria, their rapid and effective capture and killing are crucial to prevent further infection and promote osseointegration. To achieve this, we introduced fascaplysin, a β‐carboline derivative with a distinctive cationic five‐ring coplanar scaffold, to modify TNA. The cationic moieties in the molecular architecture of the fascaplysin derivative 14 (F‐14) enable precision targeting of bacterial membranes through electrostatic interactions with negatively charged phospholipids. These cationic moieties function similar to molecular “catchers” that selectively bind and disrupt pathogens while sparing host cells. Based on our previous study, we developed a series of novel fascaplysin derivatives through systematic structural modifications.^[^ ^15^ ^]^ Preliminary antibacterial screening revealed that most of the synthesized derivatives exhibited significantly enhanced potency against methicillin‐resistant *Staphylococcus aureus* (MRSA), with minimum inhibitory concentration values lower than those of the parent compound by 1 order of magnitude. Notably, in terms of antimicrobial activity, F‐14 demonstrated a tenfold improvement over vancomycin, which is the current clinical gold standard for MRSA treatment, while maintaining an excellent hemobiocompatibility profile.^[^ ^15^, ^16^ ^]^ These findings highlight F‐14 as a promising compound for addressing the critical need for new anti‐MRSA agents with enhanced capture efficacy and reduced host cytotoxicity.

The immune response to IAIs results in excessive inflammatory factors, leading to a persistent inflammatory phase and an imbalance of bone metabolism.^[^ ^17^ ^]^ Therefore, alleviating inflammation to balance bone metabolism remains a critical need in the process of addressing IAIs after bacterial clearance. In this context, TNA, which has a microstructure similar to that of bone tissue, has demonstrated efficacy in bone repair through the modulation of macrophage polarization from an M1 inflammatory phenotype to an M2 healing phenotype. This modulation can effectively reshape the inflammatory response, stimulate the expression of osteogenesis factors, and inhibit the generation of osteoclasts.^[^ ^6^ ^]^ Thus, an effective therapy involves eradication of the pathogen and rapid modulation of the immune microenvironment following sterilization.

**Scheme 1:**
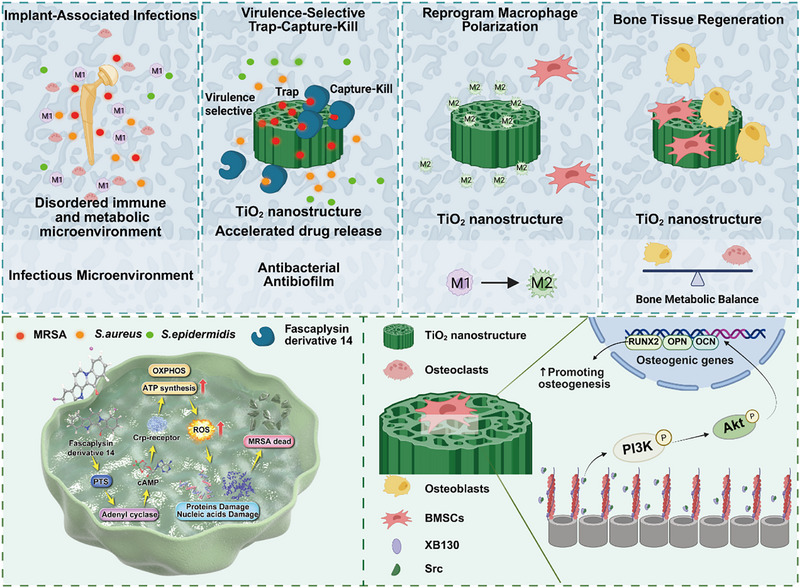
Schematic illustration of Virulence‐Selective Trap‐Capture‐Kill Antibacterial Nanostructures for effectively combating IAIs to promote local bone tissue regeneration.

In this study, we propose a virulence‐selective trap–capture–kill antibacterial system for the treatment of IAIs (**Scheme** **1**). In this system, TNA and F‐14 were designed to utilize the chemotaxis/physiotaxis trapping potential of TNA and the ability of F‐14 to capture bacteria using electrostatic forces. Subsequent to the capture of bacteria, F‐14 disrupts the phosphotransferase system cyclic adenosine monophosphate‐cyclic adenosine monophosphate receptor protein regulatory cascade and induces a reactive oxygen species –mediated pathway that damages bacterial macromolecules. Following this, the bacteria become inactive and are killed. In vitro and in vivo studies revealed that this modified titanium implant demonstrates multiple biocompatible functions, including the promotion of bone formation, inhibition of bone resorption, anti‐inflammatory effects, and antibacterial effects. This approach presents a unique strategy for addressing IAIs, offering a promising avenue for their prevention and treatment.

## 2 Results and Discussion

### 2.1 Fabrication and Characterization of fascaplysin derivative 14‐modified TiO_2_ nanostructure (F‐14‐TNA)

**Figure 1:**
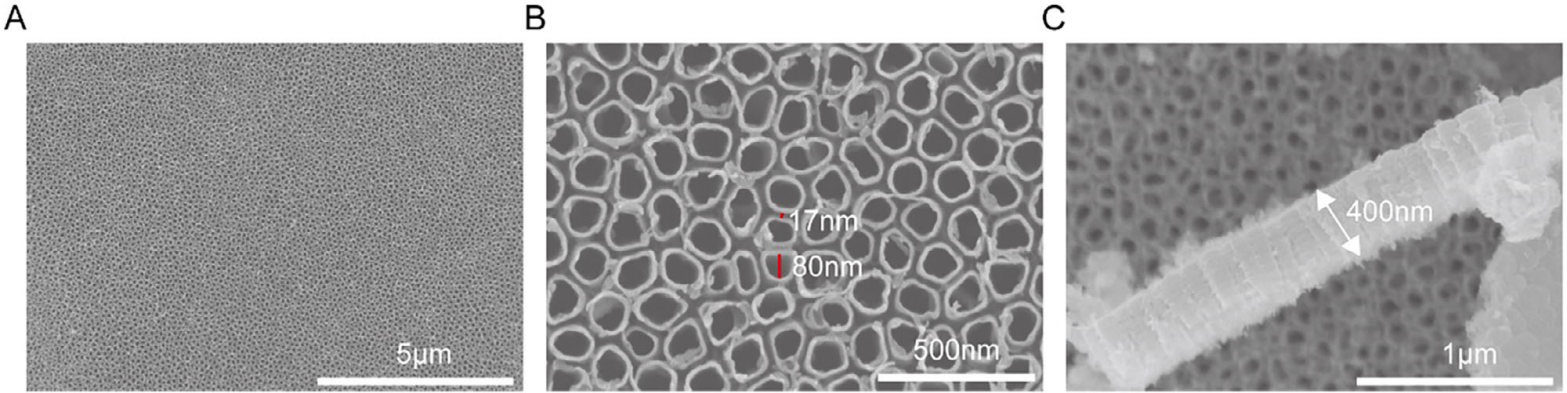
SEM images of TNA. A) Top view of the TNA; B) highly magnified top view of the TNA surface displays pore size of ≈80 nm, spacing of ≈17 nm; C) a cross‐sectional perspective reveals tube lengths of ≈400 nm.

Antibiotics are usually injected before arthroplasty to prevent periprosthetic joint infection.^[^ ^18^ ^]^ For patients with methicillin‐resistant *Staphylococcus aureus* (MRSA) colonization, some medical professionals recommend combining vancomycin with cefazolin.^[^ ^18^ ^]^ Injecting antibiotics usually has a systemic effect and may cause some damage to the body; However, the therapeutic effect at the surgical site is often insufficient. Hence, the most favorable strategy involves delivering the drug directly to the interface where the implant meets the tissue. In this study, we successfully developed drug‐loaded TNA, which can be directly integrated onto the surface of existing implants, thereby enhancing their therapeutic potential. The vertically aligned, fixed, high‐aspect‐ratio TNAs on the surface of the implants are constructed via anodization. **Figure** **1** shows scanning electron microscopy (SEM) images of TNA with a pore size of ≈80 nm, a spacing of ≈17 nm, and a length of ≈400 nm, which were formed at an anodization voltage of 20 V for 40 min. The TNA is uniformly distributed across the substrate. Our previous studies revealed that pore size is directly related to the anodization voltage, allowing the creation of substrates with various size scales by adjusting the voltage and duration of anodization.^[^ ^19^, ^20^, ^21^, ^22^ ^]^ Studies indicate that the length of TNA can be altered from 200 to 500 nm, and the pore diameter of the nanotubes can vary between 55 nm and 570 nm.^[^ ^19^, ^20^, ^21^, ^22^ ^]^ Therefore, the large specific surface area, precisely adjustable pore size, and length for optimal biotemplating activities are key advantages of TNA. These features make it highly suitable for use as a drug‐eluting implant.

In this study, we loaded an antibacterial drug called F‐14 into TNA on the surface of titanium (Ti) rods. F‐14 is a pentacyclic quaternary ammonium salt with a unique cationic five‐ring coplanar backbone that inhibits a variety of bacterial activities, particularly those of MRSA, with a minimum inhibitory concentration value of 0.098 µg mL^−1^ (10 times lower than that of vancomycin).^[^ ^15^ ^]^

**Figure 2:**
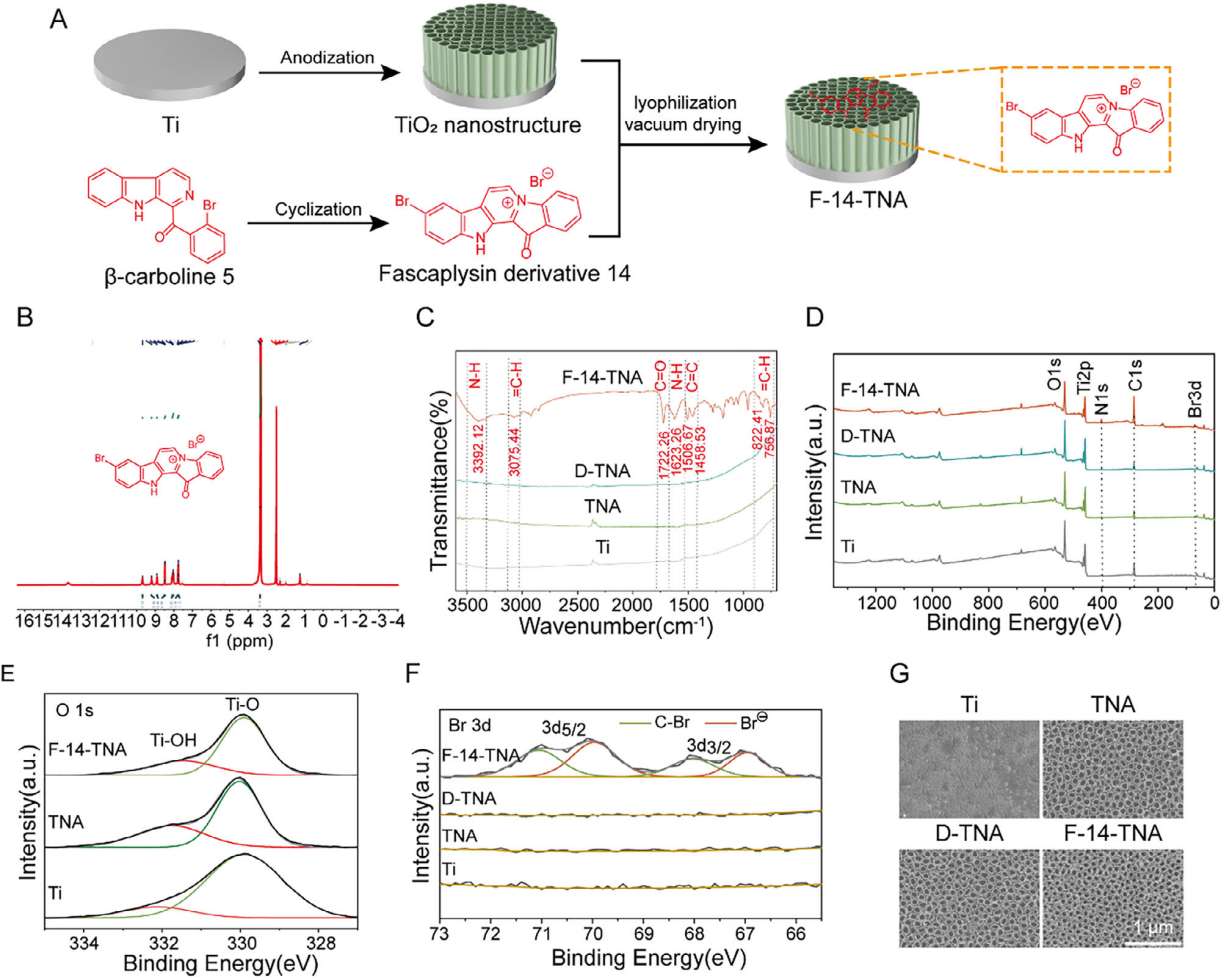
Fabrication and structural characterization of F‐14‐TNA. A) Fabrication diagram of F‐14‐TNA. B) ^1^H NMR spectra of F‐14. C) FTIR spectra of Ti, TNA, D‐TNA, and F‐14‐TNA. D) XPS analysis of Ti, TNA, D‐TNA, and F‐14‐TNA. E) High‐resolution O 1s XPS spectra of Ti, TNA, and F‐14‐TNA. F) Br 3d XPS spectra of Ti, TNA, D‐TNA, and F‐14‐TNA. G) Micromorphology observation of different sample surfaces via SEM.

In this work, F‐14‐loaded TNAs were fabricated on pure Ti by anodization, lyophilization, and vacuum drying (**Figure** **2**A). H nuclear magnetic resonance (^1^H NMR) was used to confirm the successful fabrication of F‐14 (Figure 2B). As depicted in the fourier transform infrared (FTIR) spectra (Figure 2C), characteristic bands of F‐14 appeared at 3392.12 cm^−1^ and 1623.26 cm^−1^ (m; ν(N‐H)), 3075.44 cm^−1^, 822.41 cm^−1^ and 756.87 cm^−1^ (m; ν(= C‐H)), 1722.26 cm^−1^ (m; ν(C = O)), 1506.67 and 1458.53 cm^−1^ (m; ν(C = C)). X‐ray photoelectron spectroscopy (XPS) analysis (Figure 2D) revealed that the F‐14‐TNA group had a distinct Br peak compared with the Ti, TNA, and D‐TNA groups, which indicates that F‐14 was successfully loaded into the TNA. As shown in the high‐resolution Br 3d XPS spectra (Figure 2F), the deconvoluted XPS spectra of Br 3d demonstrated two dominant peaks, characteristic of the Br 3d5/2 (at ∼955.5 eV) and Br 3d3/2 (at ∼933.5 eV) peaks. The left subpeaks (green lines, peaks at 71 and 76 eV) are characteristic of C‐Br, whereas the right subpeaks (red lines, peaks at 70 and 67 eV) can be attributed to Br^Θ^. The O 1s spectrum can be divided into two peaks: the peak at 531.6 eV can be assigned to the Ti‐OH species, and the peak at 529.9 eV corresponds to the Ti‐O species (Figure 2E). Figure 2G shows the SEM surface morphologies. Compared with those of the Ti, TNA, and D‐TNA groups, the surface morphologies of the F‐14‐TNA group did not significantly differ. Furthermore, EDS mapping images revealed that F‐14 was uniformly loaded into the entire TNA (Figure S1, Supporting Information). Hence, loading F‐14 in TNAs and using this TNA as a drug‐eluting platform is an effective way to deliver this drug.

### 2.2 Properties of F‐14‐TNA

**Figure 3:**
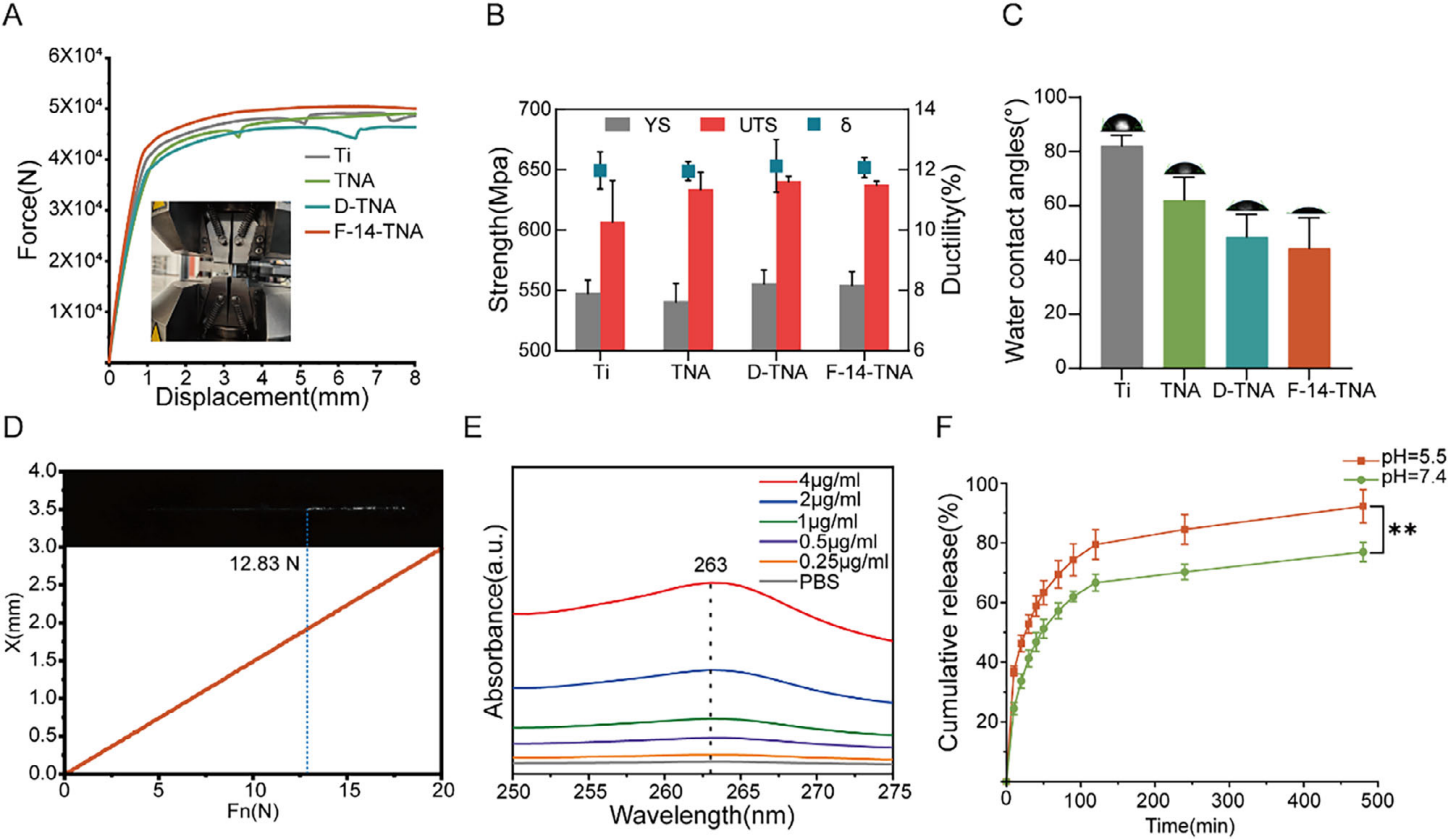
Mechanical properties, hydrophilicity, and F‐14 release rate of F‐14‐TNA. A) Tensile force and displacement curves of Ti, TNA, D‐TNA, and F‐14‐TNA. B) Yield strength (YS), ultimate tensile strength (UTS), and elongation (δ) of Ti, TNA, D‐TNA, and F‐14‐TNA. C) Water contact angles of Ti, TNA, D‐TNA, and F‐14‐TNA. D) Scratch test of F‐14‐TNA. E) UV absorption spectra of F‐14. F) Cumulative release of F‐14 from F‐14‐TNA in different pH solutions. Data represent the mean ± SD; n = 3; ** *p* < 0.01.

The mechanical properties of F‐14‐TNA were evaluated via a universal material testing machine (Instron 5969; USA) at room temperature. **Figure** **3**A shows the tensile force and displacement curves of Ti, TNA, D‐TNA, and F‐14‐TNA, and Figure 3B shows the yield strength (YS), ultimate tensile strength (UTS), and elongation (δ) of the Ti, TNA, D‐TNA, and F‐14‐TNA groups. These results showed that the mechanical properties of F‐14‐TNA did not change compared with those of the Ti group. Anodization can prepare well‐controlled nanostructures on the surface of titanium materials without affecting the overall mechanical properties of the Ti material, which would other be impossible.^[^ ^23^ ^]^

Water contact angles were measured to evaluate the surface wettability of F‐14‐TNA (Figure 3C), and Ti, TNA, and D‐TNA were used as the control groups. The water contact angles on the surfaces of Ti, TNA, D‐TNA, and F‐14‐TNA were 82.2°, 62.1°, 48.6°, and 45.5°, respectively. The construction of TNA, whose surface contains hydroxyl groups, significantly reduces the contact angle of the surface of TNA, D‐TNA, and F‐14‐TNA and increases their hydrophilicity.^[^ ^24^, ^25^ ^]^ The F‐14‐TNA surface has the lowest water contact angle and the best hydrophilic properties. Our previous studies revealed that the hydrophilic surface of TNA could promote cell adhesion.^[^ ^19^ ^]^ Between the sample surface and the cell wall proteins. The improvement in hydrophilicity may promote cell adhesion between the matrix and cell wall proteins and select more virulent bacterial strains by inducing the adhesion and entrapment process.^[^ ^26^ ^]^

The mechanical strength of the functional layer on the surface of the implant is of paramount importance, as it directly influences the long‐term stability of the implant.^[^ ^24^ ^]^ This strength should be sufficient to withstand the rigors of the implantation process, ensuring durability and reliability over time. Consequently, we conducted a nano‐scratch test to assess the adhesion strength of the F‐14‐TNA layer on the titanium surface. The critical load of the functional layer achieved 12.83 N (Figure 3D). This outcome indicates that the functional layer, namely F‐14‐TNA, demonstrated excellent bonding strength with the titanium surface. Such robust adhesion is crucial for ensuring the mechanical stability of implants during surgical procedures.

The release rates of F‐14 from F‐14‐TNA were investigated via UV absorption spectroscopy. To simulate the acidic microenvironment of the IAIs (pH = 5.5) and normal physiological environment (pH = 7.4), the UV absorption spectra at a wavelength of 263 nm in pH 5.5 and pH 7.4 buffer solutions (Figure 3E, Figure S2, Supporting Information), respectively, were measured to conduct the F‐14 release tests. As shown in Figure 3F, in the acidic microenvironment, 92.3 ± 5.6% of the F‐14‐TNA was released within 480 min, which was significantly greater than that in the normal physiological environment (76.9 ± 3.2%). This phenomenon may be attributed to the competitive interaction of positively charged protons and F‐14 with the surface charge of the TNA interface. In the microenvironment of bacterial infection, small‐molecule protons with a positive charge exist. F‐14 is a cationic structure with a positively charged surface, and TNA has a surface composed of terminal hydroxyl groups, leading to a slight negative charge on the surface.^[^ ^15^, ^27^ ^]^ Thus, positively charged small molecule protons are more likely to interact with the TNA surface charge than positively charged F‐14, probably due to the stronger electrostatic interaction between the protons and the titanium dioxide surface.^[^ ^15^, ^25^, ^27^ ^]^

### 2.3 Bacterial Virulence‐Selective Trap‐Capture‐Kill and Antibacterial Evaluation of F‐14‐TNA In Vitro

**Figure 4:**
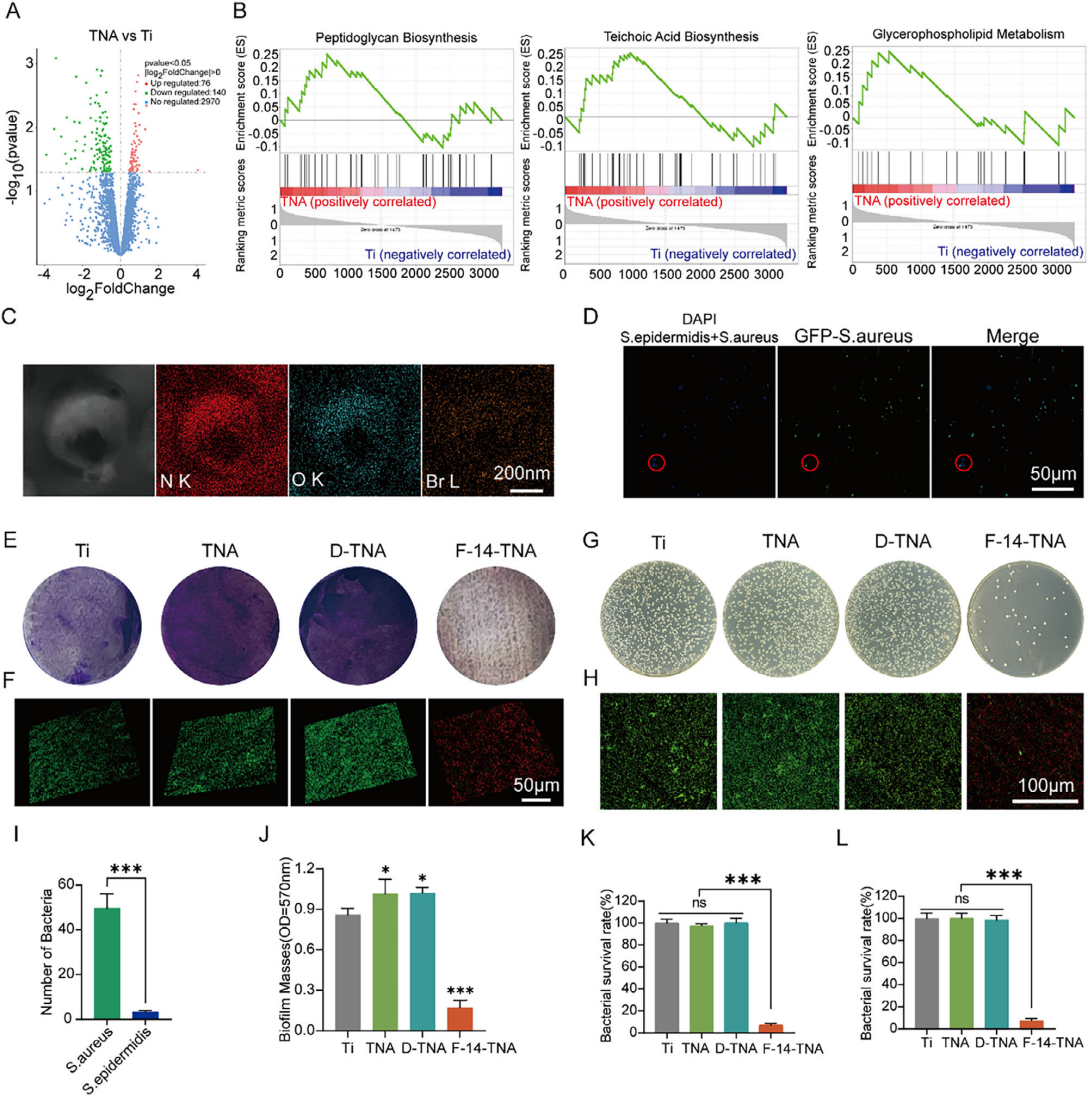
Bacterial virulence‐selective trap‐capture‐kill and antibacterial evaluation of F‐14‐TNA in vitro. A) DEGs in bacteria in the TNA and Ti groups. B) GSEA of peptidoglycan biosynthesis, teichoic acid biosynthesis, and glycerophospholipid metabolism in bacteria in the TNA and Ti groups. C) TEM images and elemental mapping of MRSA treated with F‐14‐TNA. D,I) CLSM images of *S. aureus* and *S. epidermidis* were co‐cultured with TNA and corresponding bacterial numbers (Red circles: Differences between *S. aureus* and *S. epidermidis* on the TNA surface). E,J) Crystal violet staining of MRSA biofilms following various treatments and corresponding quantitative plots. F) 3D CLSM Live/Dead staining images of MRSA biofilms following various treatments. G,K) Photographs of MRSA planktobacterial colonies formed on LB agar plates with various treatment groups and the corresponding planktobacterial survival rates. H,L) Live/Dead CLSM images of MRSA treated with Ti, TNA, D‐TNA, and F‐14‐TNA and the corresponding planktonic bacterial survival rates. The data represent the mean ± SD; n = 3; * < 0.05, *** *p* < 0.001.

Bacterial colonization is essential for the development of peri‐implant infection.^[^ ^28^ ^]^ When bacteria adhere to the implant's surface before the host cells, the bacteria adhere to each other quickly and accumulate to form a dense biofilm.^[^ ^29^ ^]^ To investigate the potential biological effects of TNA toward MRSA, the mRNA transcriptomes of MRSA treated with Ti and TNA were analyzed. Based on the mRNA sequencing results, the differences in gene expression between the TNA group and the pure Ti group are shown in **Figure** **4**A. The differentially expressed genes (DEGs) were obtained by treating MRSA with TNA as the experimental group, which were identified by |log_2_FoldChange| ≥ 0 and padj ≤ 0.05. As shown in Figure 4A, a total of 216 DEGs were observed in the TNA and Ti groups. A gene set enrichment analysis (GSEA) was conducted on the molecular signature database (MSigDB). We found that the DEGs were enriched mainly in pathways associated with peptidoglycan biosynthesis, teichoic acid biosynthesis, and glycerophospholipid metabolism signaling (Figure 4B). Peptidoglycan and teichoic acid are the main components of the cell wall of Gram‐positive bacteria,^[^ ^30^, ^31^ ^]^ while glycerophospholipids are the main components of the cell membrane. Transcriptome results showed that TNA provided an ideal platform for bacteria to adhere, proliferate, and aggregate. Therefore, the utilization of TNA as a trap agent to recruit bacteria for further elimination is a promising strategy. As shown in Figures S3 and S4, Supporting Information, TNA group surfaces were able to attract more MRSA than the Ti group, which was due to the favorable superficial chemical environment and the nanocavities of TNA, which enabled entrapment of bacteria. In addition, the positively charged nature of F‐14 allows it to precisely locate and bind to bacteria like a “catcher.” As shown in the mapping results in Figure 4C, Br accumulates on the surface of bacteria in F‐14‐treated MRSA. The BODIPY‐TR‐cadaverine fluorescence stain further provided strong evidence that F‐14 has binding affinity for anionic LTA present in bacterial membranes (Figure S5, Supporting Information). This observation suggests that the initial attachment of F‐14 to the cell wall is facilitated by electrostatic attraction, highlighting the key role of charge interactions in this binding event. Thus, a trap‐capture‐kill antibacterial system was hypothesized. Within this system, TNA served as a trap‐attractant to facilitate bacterial recruitment, while F‐14 functioned as a capture agent to facilitate bacterial adsorption. Subsequently, the bacteria were killed under sustained F‐14 release.

The virulence of certain bacterial lineages is contingent upon their adhesion capabilities, invasiveness, and exocrine characteristics. Here, we selected a combination of hyperpathogenic bacteria (GFP‐labeled *S. aureus*) and hypopathogenic bacteria (*S. epidermidis*), which are commonly found in the clinical environment, as the model combination of infectious exposures. As shown in Figure 4D,I and *S. aureus* and *S. epidermidis* were co‐cultured with TNA for 24 h, confocal laser scanning microscopy (CLSM) observation showed that S. aureus (GFP) adhered to the TNA surface much more than *S. epidermidis*. Further validation through bacterial aggregation and trap experiments.^[^ ^32^ ^]^ Compared with the Ti group, TNA, D‐TNA, and F‐TNA all exhibited significant bacterial trap effects against MRSA and *S. aureus*, causing bacterial aggregation. At the same time, TNA, D‐TNA, and F‐TNA had no significant effect on *S. epidermidis* (Figure S6, Supporting Information). These phenomena may be attributed to that high virulence bacterial colonies are much more likely to adhere and later invade deeper tissue. We introduced nanostructure to the surface to trap the highly virulent bacterial strains. We discovered both bacterial virulence selectivity in the same strain, as demonstrated in RNA‐seq, and a mixture of different pathogenic bacterial strains. These results indicate that TNA is selective and preferentially traps highly pathogenic bacteria, paving the way for preferentially killing highly pathogenic bacteria in the future.

In the past decade, antibiotics have been the predominant medications employed in the treatment of bacterial infections, serving as a cornerstone in the fight against these ailments. However, the biofilm formation on the surface of orthopedic implants causes physical barriers to the penetration of antibiotics and the host immune system, leading to a large increase in antibiotic‐resistant bacteria, causing more serious health problems.^[^ ^33^ ^]^ Crystal violet staining and 3D CLSM images of Live/Dead staining were employed to evaluate the effectiveness of F‐14‐TNA in eradicating biofilms. As shown in Figure 4E–J, compared with the Ti group, the biofilm formation of MRSA treated with F‐14‐TNA was significantly reduced.

To prevent the reformation of eradicated biofilms, it is of paramount importance to eliminate the planktobacteria released from the microenvironment of eradicated biofilms.

Since MRSA is the most common multidrug‐resistant bacterium causing postoperative infections, F‐14‐TNA eliminated the planktobacteria evaluation used MRSA as a model bacterium.^[^ ^34^, ^35^ ^]^ F‐14‐TNA elimination of the planktobacteria was assessed via spread plates, and in vitro bacterial survival was further verified via Live/Dead fluorescence staining. MRSA in the logarithmic growth phase was cocultured with four groups (Ti, TNA, D‐TNA, and F‐14‐TNA). Following a 24‐h incubation at 37 °C in an incubator, the bacterial suspensions of each group were subjected to an agar medium spread plate, and then the colonies were quantified. Compared with those of the Ti, TNA, and D‐TNA groups, the residual bacterial colonies of the F‐14‐TNA groups decreased significantly (Figure 4G,K). Moreover, the Live/Dead fluorescence staining assay confirmed the presence of significantly redder fluorescent‐stained (dead) bacterial cells in the F‐14‐TNA group than in the Ti, TNA, and D‐TAN groups, which contained predominantly live, green fluorescent‐stained bacteria (Figure 4H,L). Thus, F‐14‐TNA has an excellent eradicated planktonic bacteria effect.

The above results indicate that TNA acts as a passive “trap” with a nanostructure that selectively enriches highly virulent strains by enhancing bacterial adhesion, but lacks bactericidal function; D‐TNA acts as a solvent (DMSO) control with negligible antimicrobial activity, confirming the central role of F‐14; and F‐14‐TNA innovatively integrates virulence selective trap‐ capture‐kill multiple actions.

### 2.4 Antibacterial Mechanism of F‐14‐TNA

**Figure 5:**
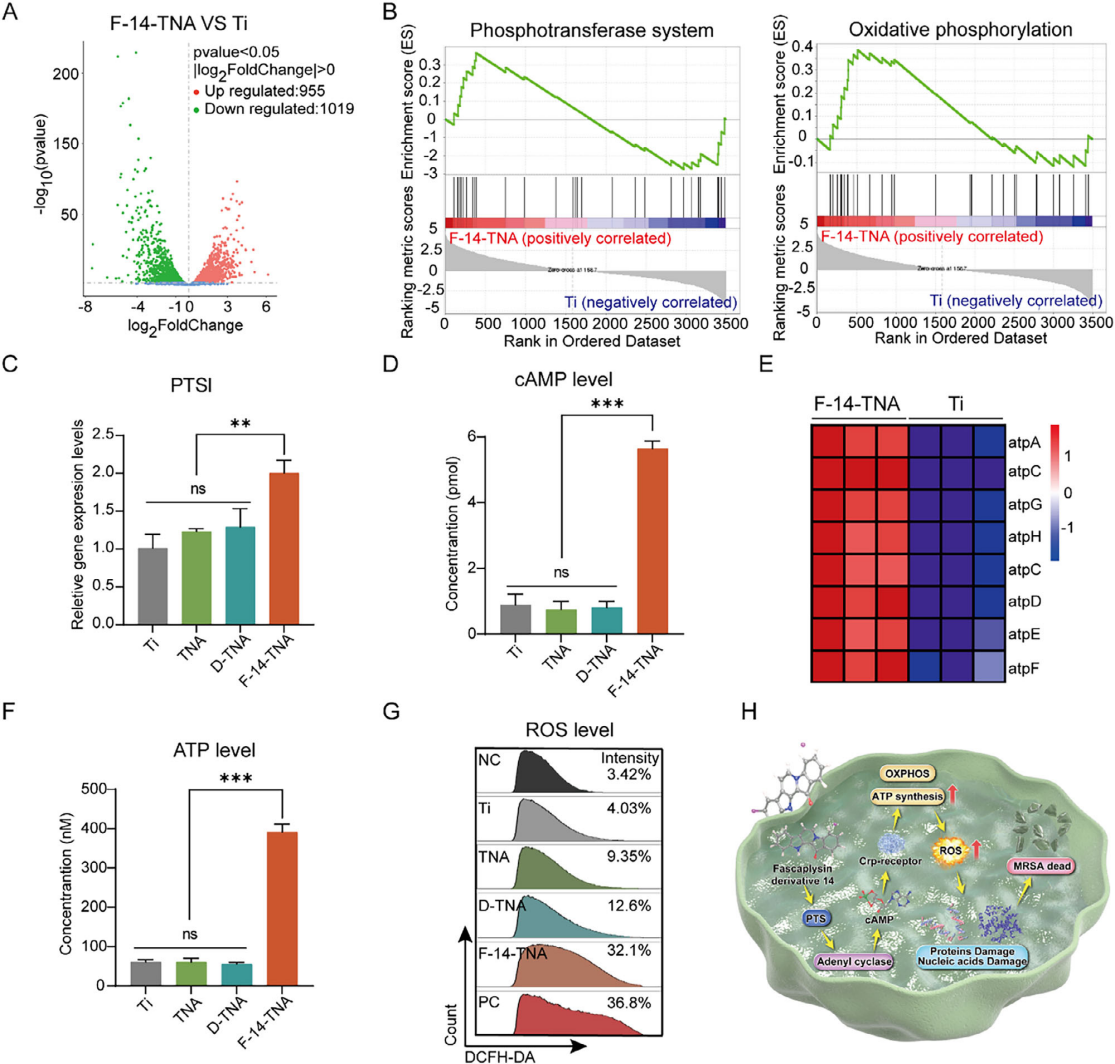
Antibacterial mechanism of F‐14‐TNA. A) Volcano plot of DEGs in bacteria in the F‐14‐TNA and Ti groups. B) GSEA of phosphotransferase system and oxidative phosphorylation in bacteria in the F‐14‐TNA and Ti groups. C) mRNA expression levels of the PTSI gene in bacteria cultured on various samples. D) Intracellular cAMP levels in bacteria in the Ti, TNA, D‐TNA, and F‐14‐TNA groups. E) Heatmap of gene expression in bacteria in the F‐14‐TNA and Ti groups. F) Intracellular ATP levels in bacteria in the Ti, TNA, D‐TNA and F‐14‐TAN groups. G) Intracellular ROS levels in bacteria in the NC, Ti, TNA, D‐TNA, F‐14‐TNA and PC groups. NC: negative control group; PC: positive control group. (H) Schematic representation of the antibacterial mechanism in vitro. The data represent the mean ± SD; n = 3; ** *p* < 0.01, *** *p* < 0.001.

To investigate the potential antibacterial mechanism of F‐14‐TNA toward MRSA, the mRNA transcriptomes of MRSA treated with Ti, TNA, D‐TNA, and F‐14‐TNA were analyzed. Based on the mRNA sequencing results, the differences in gene expression between the F‐14‐TNA group and the pure Ti, TNA, and D‐TNA groups are shown in **Figure** **5**A, and Figures S7 and S8A,C, Supporting Information. TDEGs were obtained by treating MRSA with F‐14‐TNA as the experimental group, which were identified by |log_2_FoldChange| ≥ 0 and padj ≤ 0.05. As shown in Figure 5A and Figure S8A,C, Supporting Information, a total of 1974 DEGs were observed in the F‐14‐TNA and Ti groups, while the number of DEGs was 2270 for the F‐14‐TNA and TNA groups and 2273 for the F‐14‐TNA and D‐TNA groups. A GSEA was conducted on the MSigDB. We found that the DEGs were enriched mainly in pathways associated with phosphotransferase system (PTS) signaling (ptsI) (Figure 5B, Figure S8B,D, Supporting Information) and oxidative phosphorylation (OXPHOS) signaling (aptA, aptC, aptD, aptE, aptF, aptG, and aptH) (Figure 5E, Figure S9, Supporting Information). We speculated that F‐14‐TNA exerts its antibacterial effect against MRSA by modulating the PTS and the cyclic adenosine monophosphate‐cyclic adenosine monophosphate receptor protein (cAMP‐Crp) cascade, which transmits signals to a common reactive oxygen species (ROS)‐mediated macromolecule‐damaging pathway to inhibit bacteria.

To prove the PTS‐cAMP‐Crp cascade‐regulated antibacterial mechanism of F‐14‐TNA, we tested the expression of the ptsI gene via RT‒qPCR. As shown in Figure 5C, RT‒qPCR analyses revealed that F‐14‐TNA upregulated the expression of ptsI, a genetic study associated with tolerance to a deficiency in ptsI, a gene in the carbohydrate uptake PTS.^[^ ^36^ ^]^ This deficiency imparts tolerance to various lethal disinfectants, antibiotics, and environmental stresses. The phosphorylation cascade of the PTS elevates cAMP levels, and deficiencies in adenyl cyclase similarly impart pantolerance.^[^ ^37^ ^]^ The ELISA results also confirmed this conclusion (Figure 5D). cAMP binds to the cAMP receptor protein (Crp, cyclic adenosine monophosphate receptor protein) to form a complex known as cAMP‐Crp. cAMP‐Crp regulates the expression of oxidative phosphorylation genes (Figure 5E), promoting an increase in ATP synthesis (Figure 5F). This process is crucial for maintaining the cellular energy balance. Additionally, ATP, as the primary carrier of energy, plays a key role in modulating the cellular redox balance and the generation of ROS (Figure 5G, Figure S10, Supporting Information). Elevated levels of ROS can induce damage to bacterial macromolecules, such as proteins, nucleic acids, and lipids, ultimately leading to bacterial death (Figure 5H). TEM further demonstrated leakage of MRSA cellular contents, MRSA lysis, and membrane component loss (Figure S11, Supporting Information). The above results validate our hypothesis. Specifically, it is believed to involve the interaction between the positively charged quaternary ammonium groups of F‐14 and the negatively charged phosphoryl groups of the phospholipid components of bacterial membranes. This interaction affects the integrity of the cytoplasmic membrane, ultimately causing cell death.^[^ ^15^, ^38^, ^39^ ^]^

### 2.5 In Vitro Evaluation of Biocompatibility

There are various applications for TNA on implants. However, applying TNA for orthopedic implants is the main focus of this study. Thus, we investigated the functionality of BMSCs on TNA surfaces. Because favorable biocompatibility is the most basic requirement for biomedical materials,^[^ ^28^, ^40^, ^41^ ^]^ we performed CCK‐8 assays to evaluate cell viability and proliferation on the surface of F‐14‐TNA and investigated the effect of drug release on cell viability and proliferation. The results showed that the cell viability and proliferation on TNA, D‐TNA, and F‐14‐TNA surfaces were significantly higher than those on pure Ti surfaces on day 7, probably due to the nanomorphology. Notably, at the early time points, day 1 and day 3, there was no significant difference in cell viability and proliferation rate on the surface of F‐14‐TNA compared with the other three groups (Figure S12A, Supporting Information). To further evaluate cytotoxicity, live and dead cell staining and cytoskeleton staining were performed; the results indicate that the viability of BMSCs on the Ti, TNA, D‐TNA, and F‐14‐TNA surfaces was not reduced, and the MC3T3‐E1 preosteoblasts exhibited normal morphology with a triangular or spindle, indicating that F‐14‐TNA had no obvious cytotoxicity (Figures S12C and S13, Supporting Information). In addition, as a biomedical implant, F‐14‐TNAs inevitably contact blood, so blood compatibility is also a basic requirement for biomedical implants.^[^ ^42^ ^]^ As shown in Figure S12B, Supporting Information, the positive control group (Triton X‐100) is red, whereas the Ti, TNA, D‐TNA, and F‐14‐TNA groups appear colorless, similar to the negative control group (0.9% normal saline). The hemolysis ratios were 0.62%, 0.43%, 0.24%, and 0.19% for the Ti, TNA, D‐TNA, and F‐14‐TNA groups, respectively. The hemolysis rates of the Ti, TNA, D‐TNA, and F‐14‐TNA groups were less than 2%, indicating the outstanding blood compatibility of the F‐14‐TNA.^[^ ^43^ ^]^ These findings suggest that F‐14‐TNA has excellent cytocompatibility in vitro.

### 2.6 In Vitro Osteogenic Property Evaluation of F‐14‐TNA

**Figure 6:**
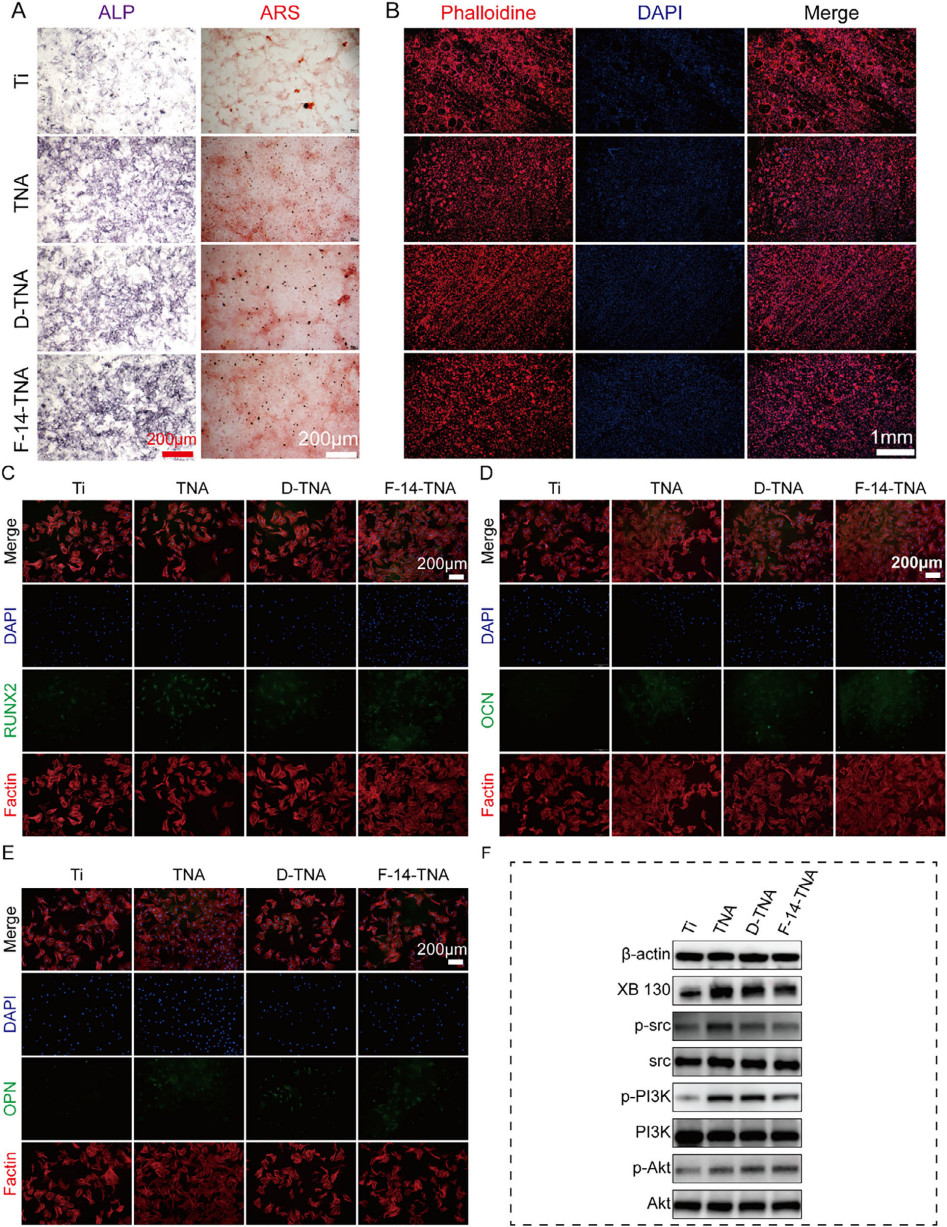
In vitro osteoclast inhibition and osteogenic differentiation evaluation of F‐14‐TNA. A) Osteogenic differentiation of BMSCs treated with various groups by ALP at 7 days and ARS at 21 days. B) Phalloidin‐labeled osteoclast cytoskeleton. C–E) IF analysis revealed RUNX2, OCN, and OPN expression in BMSCs cells. F) WB analysis revealed XB130, p‐Src, p‐PI3K, and p‐Akt expression in BMSCs cells. N = 3.

Ideally, Orthopedic implants should not only reveal excellent antibacterial properties but also exhibit good osteogenic and osteoinductive properties.^[^ ^44^ ^]^ To evaluate the effect of F‐14‐TNA on the osteogenic properties of BMSCs, we examine the alkaline phosphatase (ALP) activity and calcium phosphate deposition, and the expression of critical marker proteins. ALP staining revealed markedly elevated ALP activity in BMSCs cultured on TNA, D‐TNA, and F‐14‐TNA group surfaces for 7 days (**Figures** **6**A, and S14A, Supporting Information). ARS staining was used to identify calcium phosphate deposition, which was considered an indicator of bone regeneration, as illustrated in Figures 6A and S14A, Supporting Information. The TNA, D‐TNA, and F‐14‐TNA groups enhanced mineralized nodule formation compared to the smooth Ti surface. The expression of OCN, OPN, and RUNX2 was evaluated by immunofluorescence (IF) staining after 7 days of co‐culture (Figure 6C–E, Figure S14C, Supporting Information). The results showed that TNA, D‐TNA, and F‐14‐TNA significantly increase RUNX2, OCN, and OPN relative to the Ti group. These results indicate that the TNA facilitate in vitro osteogenesis.

TNA topography, acting as a form of nanomechanical stimulation, markedly enhances osteogenesis and augments osteointegration at the implant‐bone interface.^[^ ^14^ ^]^ Related studies have shown that the improved osteogenesis of TNA may be partially attributed to mechano‐biochemical signal transduction mediated by filamentous actin and XB130. XB130, a member of the actin filament‐associated protein family, functions as an adaptor protein, modulating both cytoskeletal dynamics and tyrosine kinase‐mediated signaling pathways. XB130 modulates the osteogenic differentiation of BMSCs through the Src and PI3K/Akt signaling cascades. Therefore, we hypothesize that F‐14‐TNA promotes osteogenesis probably through the interaction of XB130 with Src, which leads to the activation of the downstream PI3K/Akt pathway, thereby promoting bone regeneration and osseointegration. To prove our assumption, the expression levels of XB130, Src, PI3K, and Akt were evaluated by WB after 7 days of co‐culture. The results showed that TNA, D‐TNA, and F‐14‐TNA significantly increase XB130, Src, PI3K, and Akt relative to the Ti group (Figure 6F).

Fluorescence staining was employed to assess the impact of the various samples on osteoclast formation. F‐actin rings are a marker of osteoclast activity and reflect the integrity of their cytoskeleton. The Ti group had more F‐actin rings than the other groups. Results showed that TNA inhibits osteoclast formation (Figure 6B, Figure S14B, Supporting Information).

These results suggest that TNA has a dual effect of promoting osteogenesis and inhibiting osteoclast formation.

### 2.7 Reprogram Macrophage Polarization

Long‐term IAIs trigger a sustained inflammatory response, potentially compromising bone regeneration. Macrophage function, modulated by polarization, is critical in orchestrating immune responses across distinct phases of tissue repair, thereby influencing the outcome of bone healing.^[^ ^45^ ^]^ Within the context of infected bone loss, the accrual of pro‐inflammatory M1 macrophages is associated with inflammation, while M2‐like macrophages are critical to bone regeneration.^[^ ^46^, ^47^, ^48^ ^]^

**Figure 7:**
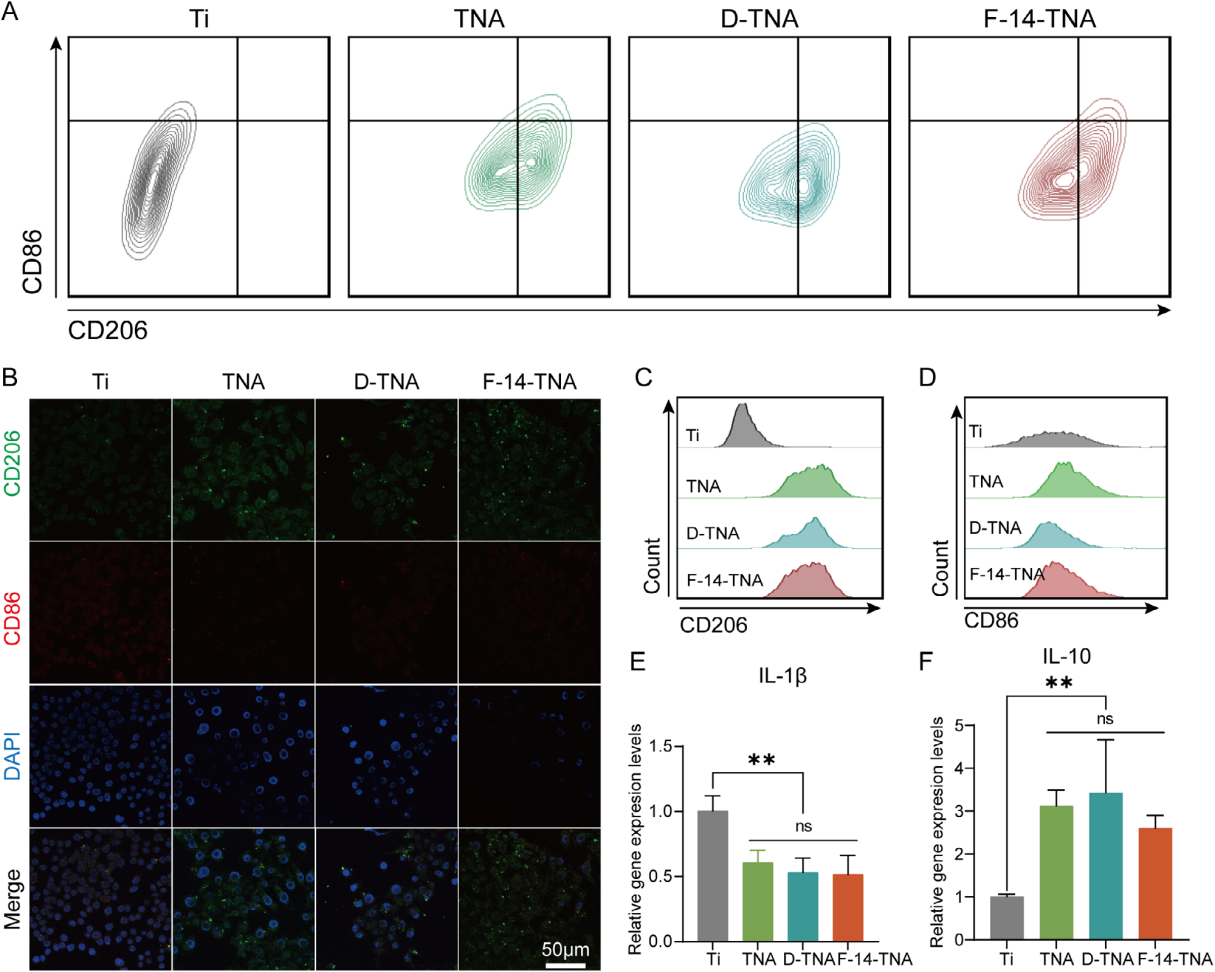
Reprogram macrophage polarization of F‐14‐TNA in vitro. A,C, D) Flow cytometric analysis of the expression levels of M1 macrophages (CD86) and M2 macrophages (CD206). B) IF analysis of the expression levels of M1 macrophages (CD86) and M2 macrophages (CD206). E,F) Relative gene expression levels of pro‐inflammatory (IL‐1β) and anti‐inflammatory genes (IL‐10). The data represent the mean ± SD; n = 3; **p < 0.01.

Flow cytometry and IF analysis assessment revealed that treatment with F‐14‐TNA resulted in a significant downregulation of inducible cluster of differentiation 86 (CD86) expression, a marker of M1 macrophages, and a corresponding upregulation of cluster of differentiation 206 (CD206) expression, a marker of M2 macrophages (**Figure** **7**A–D). To investigate the cytokine profile, we performed RT‐qPCR assays. The results of the study exhibited a marked decrease in the levels of pro‐inflammatory cytokines, specifically interleukin‐1β (IL‐1β), in the F‐14‐TNA group. Conversely, the F‐14‐TNA group exhibited a marked upregulation of anti‐inflammatory gene markers (IL‐10) (Figure 7E,F). These findings suggest that TNA components may be employed to induce macrophage polarization toward an anti‐inflammatory phenotype, thereby promoting bone regeneration.

### 2.8 In Vivo Antibacterial Evaluation of F‐14‐TNAs Against MRSA

**Figure 8:**
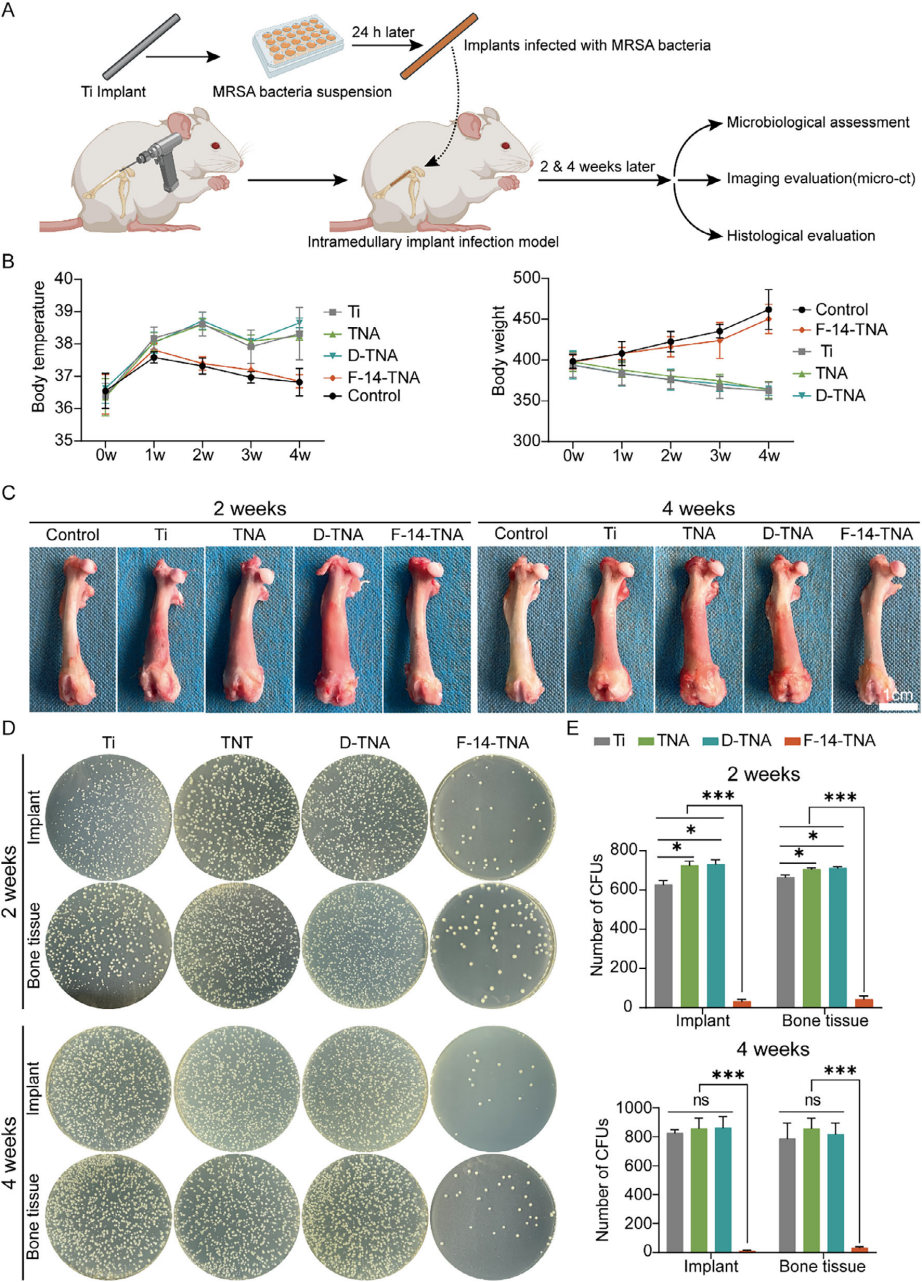
Antibacterial evaluation of F‐14‐TNA in vivo. A) Schematic diagram of the basic workflow of the in vivo experiments. B) Trends in the body temperature (°C) and body weight (g) of the SD rats in the control, Ti, TNA, D‐TNA, and F‐14‐TNA groups. C) General observations of SD rat femurs at 2 and 4 weeks. D,E) Photographs of MRSA bacterial colonies and corresponding bacterial survival rates were obtained by ultrasound treatment at 2 and 4 weeks. In the control group (uninfected group), only holes were drilled, and no metal rods were implanted. The data represent the mean ± SD; n = 3; *p < 0.05, ***p < 0.001.

Bacterial colonization of a material surface occurs in two phases: an instantaneous, reversible physical phase and an irreversible molecular and cellular phase.^[^ ^49^ ^]^ Bacterial colonization of implanted biomaterial surfaces is an essential stage in infection pathophysiology. Bacteria cannot colonize the implant surfaces quickly and are rapidly eliminated by the immune system. After phase one colonization, the surface growth of colonizing bacteria results in biofilm formation, which is important in the direct pathogenesis of IAIs. The direct contact and growth of these organisms on the biomaterial surface are crucial in biofilm formation, as they serve to anchor the entire biofilm to the surface of the biomaterial.^[^ ^27^, ^50^ ^]^ We implanted F‐14‐TNA infected with MRSA into the femoral bone marrow cavity of SD rats to assess the efficacy of the virulence‐selective trap‐capture‐kill antibacterial system, antibacterial and anti‐biofilm ability in vivo, simulating the symptoms of IAIs in orthopedics (**Figures** **8**A, and S15, Supporting Information). The control group was designated the sham operation group, which involved only drilling without implanting the metal rod. Compared with those of the F‐14‐TNA and control groups, the body temperatures of the Ti, TNA, and D‐TNA groups were markedly elevated, whereas the body weights were markedly lower during the entire implantation period (Figure 8B). The general observations of the samples of SD rat femurs are shown in Figure 8C. At 2 and 4 weeks after implant placement in the femur, the rat femoral shaft samples were congested and thickened in the Ti, TNA, and D‐TNA groups, suggesting signs of femoral infection. It is worth noting that compared with the Ti group, the rat femoral shaft samples were congested and thickened in the TNA and D‐TNA groups were obvious, which may be since TNA is more conducive to bacterial adhesion, proliferation, and aggregation than Ti, which is consistent with the Figure 4A,E, J results. However, the symptoms of femoral infection were significantly reduced in the F‐14‐TNA group, and the general morphology of the rats returned to normal 4 weeks after the operation, suggesting that the femoral infection was controlled.

At 2 and 4 weeks post‐surgery, the implants and adjacent bone tissue were collected. The bacteria attached to the implant surface and adjacent bone tissue were separated by ultrasound. The bacterial suspension was diluted and placed on an agar medium spread plate, and the antibacterial and anti‐biofilm effects were evaluated qualitatively and quantitatively. As shown in Figure 8D,E, compared with the Ti, TNA, and D‐TNA groups, the number of bacteria on the implant surface and adjacent bone tissue in the F‐14‐TNA group was significantly reduced, indicating that F‐14‐TNA could effectively eliminate surface and deep tissue bacteria in vivo. Interestingly, the number of bacteria on the implant surface and the adjacent bone tissue in the TNA and D‐TNA groups was significantly higher than that in the Ti group 2 weeks after surgery. This phenomenon may be attributed to the TiO_2_ nanostructures induce chemotactic migration of bacteria toward modified surfaces, resulting in rapid proliferation and aggregation of bacteria. The number of bacteria in the TNA and D‐TNA groups was not different from that in the Ti group after 4 weeks.

**Figure 9:**
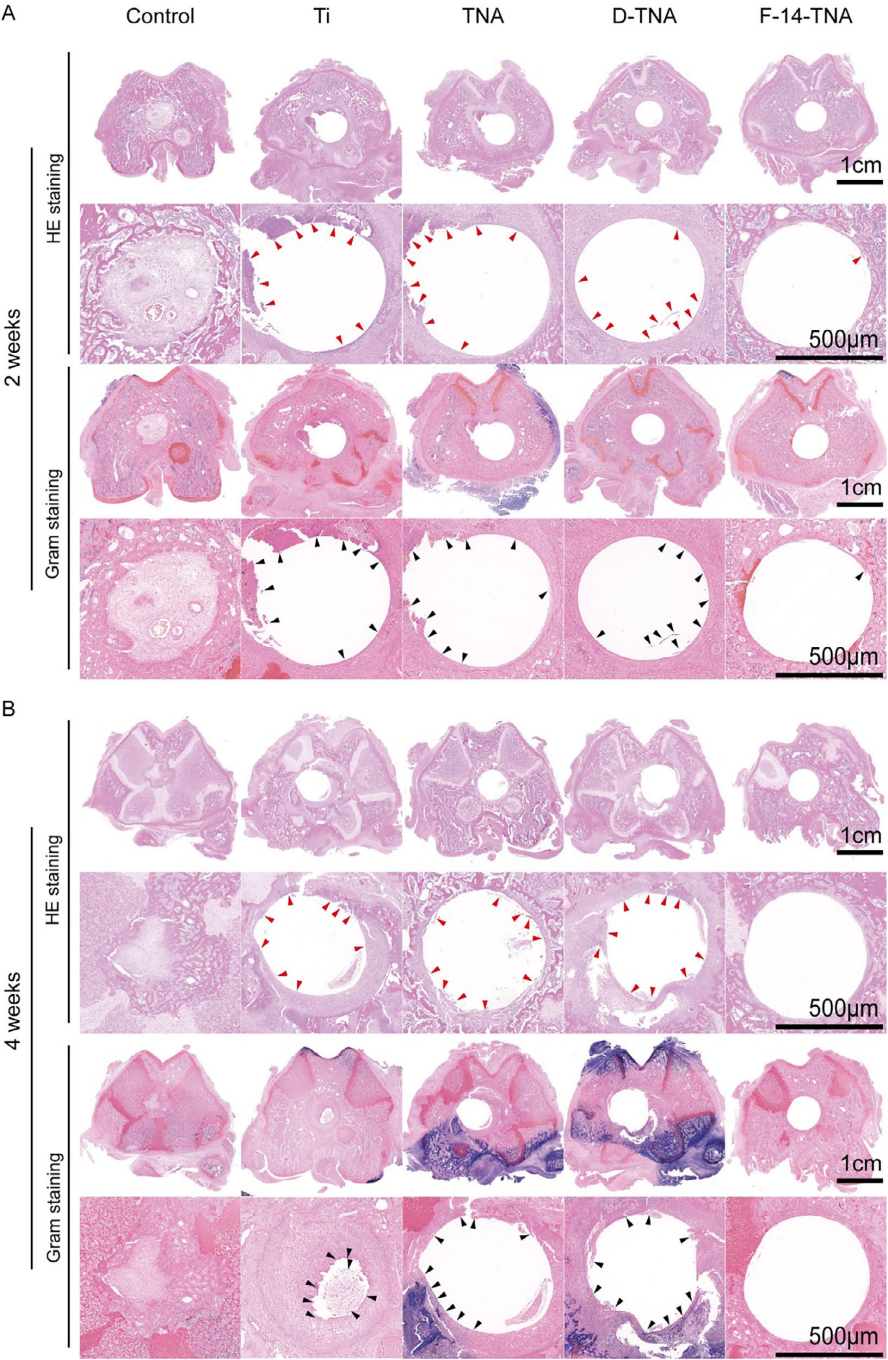
In Vivo Histopathological Analysis. A) H&E and Gram staining of peri‐implant bone tissues at 2 weeks; B) H&E and Gram staining of peri‐implant bone tissues at 4 weeks (Red arrows show inflammatory cells, and black arrows show bacteria).

H&E and Gram staining were employed to further analyze peri‐implant bone tissue for post‐implantation infection. Histological analysis with H&E and Masson staining revealed characteristic features of bone tissue infection across the Ti, D‐TNA, and F‐14‐TNA groups as evidenced by significant infiltration of monocytes and neutrophils, whereas the F‐14‐TNA group exhibited the lowest number of inflammatory cells, indicating a reduced inflammatory response (**Figure** **9**). Additionally, Gram staining further indicated minimal bacterial presence within the F‐14‐TNA group, thereby demonstrating robust anti‐biofilm efficacy within the in vivo infection model (Figure 9).

The above results show that F‐14‐TNA maintained consistent antibacterial activity throughout the study period, demonstrating its ability to prevent initial bacterial accumulation, inhibit biofilm formation, and eliminate both surface‐adherent and tissue‐invading bacteria.

### 2.9 In Vivo Osseointegration Property Evaluation of F‐14‐TNA

**Figure 10:**
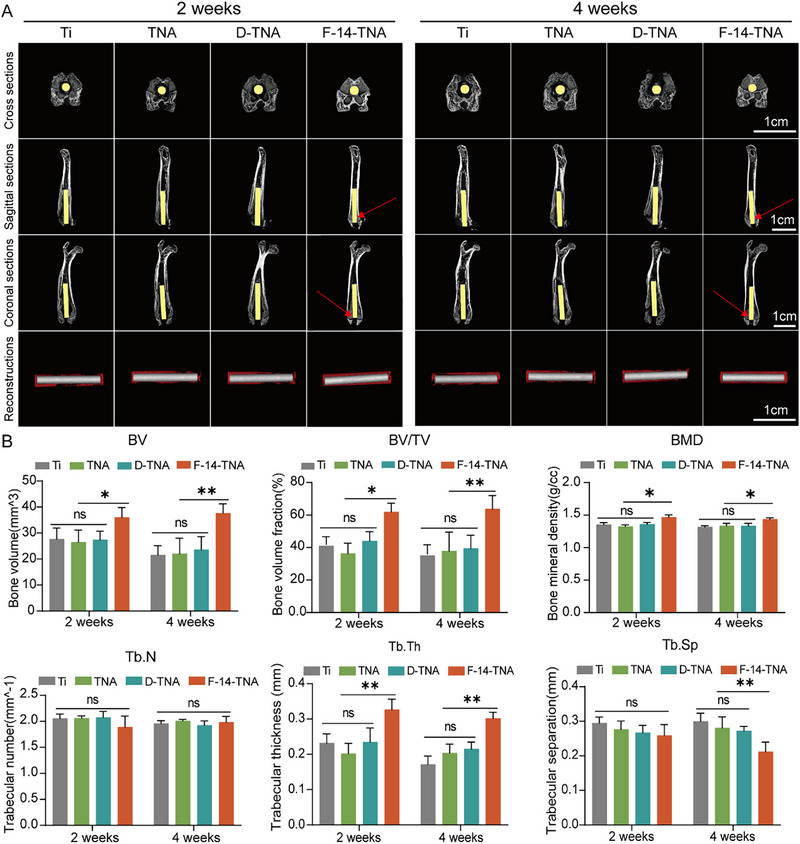
Micro‐CT assessment results. A) Cross sections, sagittal reconstructions, coronal sections, and reconstructions (yellow labels highlight the implant, red labels indicate bone tissue, and red right shows bone and implant interface integration region). B) Quantitative results of the BV, BV/TV, BMD, Tb. N, Tb. Th, and Tb. Sp in the region of the reconstructions. The data represent the mean ± SD; n = 6; *p < 0.05, **p < 0.01, and ns: not significant.

Post‐sterilization, the osteointegration of bone implant materials remains a critical consideration. Micro‐CT was employed to analyze the rat femur, assessing neo‐osteogenesis within the implant at 2 and 4 weeks following in vivo implantation. The reconstructions demonstrated significant bone resorption surrounding defects in Ti, TNA, and D‐TNA, potentially attributed to bacterial proliferation‐mediated osteolysis, whereas F‐14‐TNA displayed substantial in vivo osteogenic activity with considerable new bone formation (**Figure** **10**A). Figure 10B shows the quantitative results of BV, BV/TV, BMD, and Tb. N, Tb. Th, and Tb. Sp in the bone reconstruction regions. Compared with the Ti, TNA, and D‐TNA groups, the F‐14‐TNA group presented higher BMD, more newly formed BVs, and higher Tb. Th values and lower Tb. Sp values. No significant difference in Tb. N was observed between groups. The unchanged Tb. N suggests that F‐14‐TNA promotes bone repair primarily by thickening existing trabeculae rather than increasing trabecular quantity, which is consistent with its role in inhibiting osteolysis and enhancing mineralization. BV/TV was significantly increased and Tb. Th in the F‐14‐TNA group compared to the Ti group, confirming that it promotes bone regeneration by inhibiting osteolysis. These results indicate that the F‐14‐TNA exhibited outstanding osseointegration and antibacterial capabilities.

### 2.10 In Vivo New Bone Formation Property Evaluation of F‐14‐TNA

**Figure 11:**
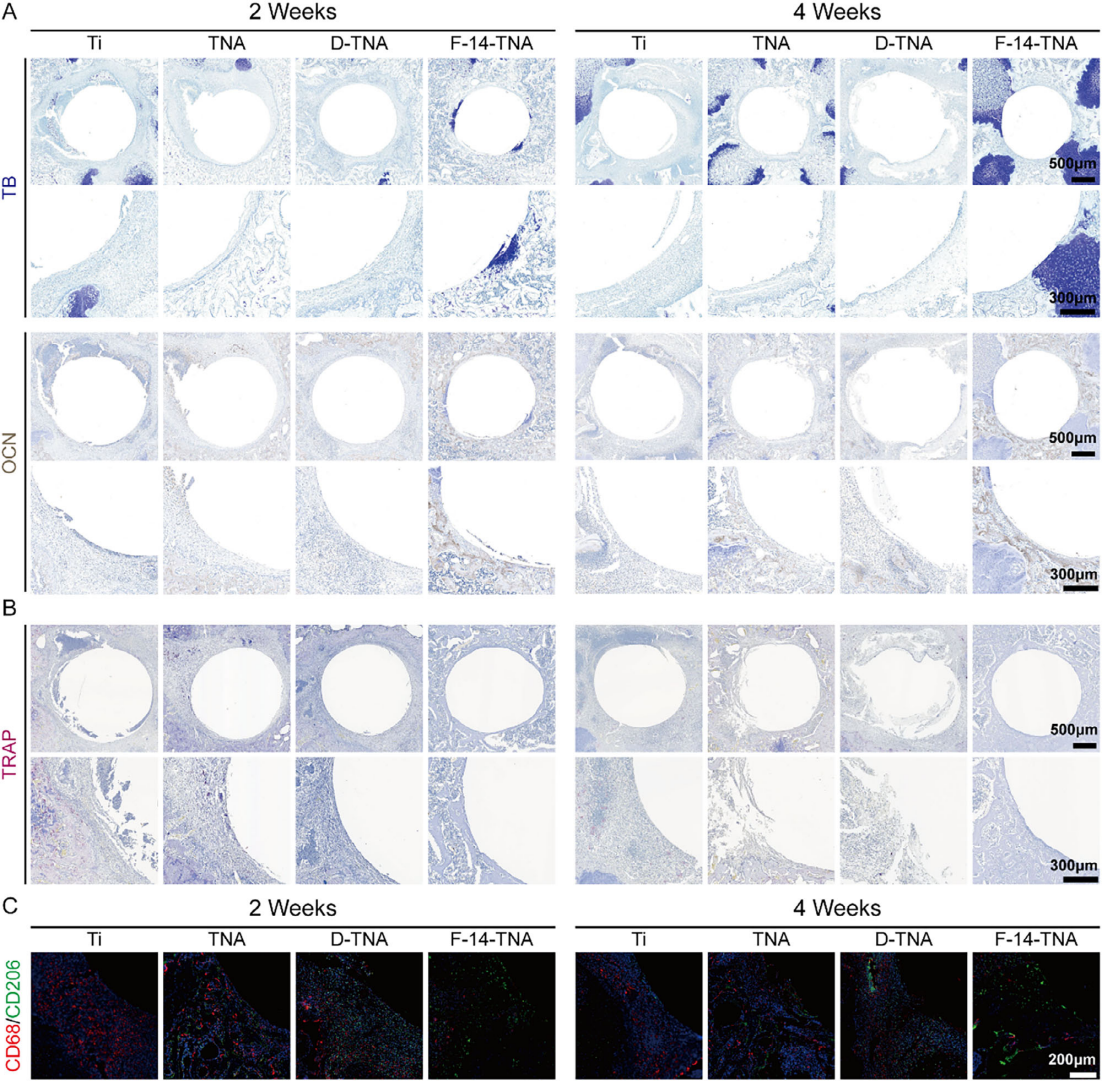
Histological and IF analyses. A) TB staining and IHC staining (OCN) of the bone tissue around the implant at postoperative 2 and 4 weeks. B) TRAP staining of the bone tissue around the implant at postoperative 2 and 4 weeks. C)  IF staining of CD68 (M1) and CD206 (M2) for macrophage polarization.

Histological evaluation included toluidine blue (TB) staining and immunohistochemical (IHC) staining (OCN) to evaluate the ability of F‐14‐TNA to promote bone formation. Masson and tartrate‐resistant acidic phosphatase (TRAP) staining were performed to assess the ability of F‐14‐TNA to prevent bone destruction (**Figure** **11**A). TB staining revealed that the F‐14‐TNA group exhibited a greater proportion of nascent endochondral ossification relative to the other group. IHC staining was employed to assess the expression of osteogenic‐related proteins (OCN) at the implant‐bone interfaces. As a marker of late osteogenesis, the expression level of OCN in the F‐14‐TNA group was higher than that in the other groups.

Infection initiation triggers a localized inflammatory response characterized by neutrophil chemotaxis, aggregation, and the release of cytokines, which subsequently activate osteoclasts.^[^ ^51^ ^]^ *S. aureus*‐derived fibronectin‐binding proteins (SpA) released during *S. aureus* infection can bind to tumor necrosis factor receptor 1 on osteoblast membranes, thereby inducing osteoblast apoptosis and suppressing osteogenic activity.^[^ ^52^ ^]^ The activation of osteoclastic activity and suppression of osteoblastic activity result in bone resorption and increase the risk of infection. Consequently, for the prophylaxis and treatment of IAIs, the implant material must exhibit osteogenic properties to effectively mitigate bone loss.

The bone destruction outcomes in rats at various time intervals are presented in Figure S16, Supporting Information. Masson staining results are displayed in Figure S16, Supporting Information; the Ti, TNA, and D‐TNA groups exhibited indications of infection and abscess formation, accompanied by bone destruction at 2 and 4 weeks post‐operation. Furthermore, high‐magnification observation revealed disorganized osteocyte distribution and fibroblast proliferation. Conversely, the F‐14‐TNA group's bone morphology appeared largely normal, with no evidence of bone destruction. Conversely, in the Ti, TNA, and D‐TNA groups, TRAP staining revealed a significant presence of abnormally activated mature osteoclasts (Figure 11B). This observation suggests that osteoclast activation occurred following the onset of osteomyelitis, with increased bone resorption worsening bone destruction and contributing to bone loss. These results show that F‐14‐TNA implants possess osteogenic properties, effectively mitigating bone destruction while simultaneously preventing infection.

Immunofluorescence staining for CD68 and CD206 was employed to assess macrophage polarization on the F‐14‐TNA implant. M1 macrophages are implicated in a pro‐inflammatory response during infection. Conversely, M2 macrophages are associated with the resolution of inflammation and the promotion of tissue repair under non‐inflammatory circumstances.^[^ ^2^ ^]^ The findings indicated a reduction in M1‐like macrophages (CD68) and an elevation in M2‐like macrophages (CD206) within the F‐14‐TNA, which implies a shift toward an M2‐like macrophage phenotype within the infected bone (Figure 11C). The result proposes that F‐14‐TNA may be capable of eliminating infection and suppressing inflammation in vivo.

While traditional antibacterial implant coatings (e.g., antibacterial agent or Ag⁺/Ga^3^⁺‐releasing surfaces) focus on broad‐spectrum bactericidal activity, they often overlook microbial heterogeneity and fail to target high‐virulence pathogens embedded in biofilms. antibacterial agent coatings (e.g., vancomycin) suffer from rapid burst release, short efficacy windows, and risk of resistance development^[^ ^53^, ^54^ ^]^; ion‐releasing coatings (e.g., Ag⁺) exhibit nonspecific cytotoxicity and poor osseointegration^[^ ^55^, ^56^ ^]^; and physical bactericidal approaches (e.g., photo/sono‐dynamic therapy) lack selectivity and may damage host tissues.^[^ ^57^, ^58^ ^]^ In contrast, our F‐14‐TNA system integrates three synergistic advantages: (1) Virulence‐selective trapping leverages the TiO_2_ nanostructure to preferentially recruit high‐virulence bacteria (e.g., *S. aureus* over *S. epidermidis*) via chemotactic adhesion (Figure 4D), addressing biofilm heterogeneity; (2) Electrostatic capture *&* cascade‐triggered killing utilizes F‐14 cationic backbone for targeted bacterial adhesion, while the PTS‐cAMP‐Crp/ROS cascade enables precise pathogen elimination without host cytotoxicity (Figure 5); and (3) Immune‐metabolic regulation uniquely promotes M2 macrophage polarization (Figure 7), balances bone metabolism (Figure 6), and enhances osteogenesis (Figures 10, 11)—transcending purely antibacterial designs to support tissue regeneration. Thus, F‐14‐TNA offers a multifunctional therapeutic platform that overcomes key limitations of conventional strategies

### 2.11 In Vivo Evaluation of Biosafety

As shown in Table S2 and Figure S17, Supporting Information, Immunocytologic results (Table S2, Supporting Information), pathological sections of vital organs in vivo (Figure S17A, Supporting Information), and serum biochemical results (Figure S17B, Supporting Information) of the F‐14‐TNA group showed no abnormalities compared with the control group. These results indicate the excellent in vivo biosafety of F‐14‐TNA.

## 3 Conclusion

In this study, we have successfully designed F‐14 modified TiO_2_ nanostructures as a virulence‐selective trap‐capture‐kill antibacterial system with superior anti‐inflammatory, pro‐osteogenic, anti‐osteoclast effects, and antibacterial effects in vitro and in vivo. According to the results of RNA sequencing, F‐14‐TNA, mainly through modulating the PTS‐cAMP‐Crp cascade, transmits signals to a common ROS‐mediated macromolecule‐damaging pathway to kill bacteria. In summary, the virulence‐selective trap‐capture‐kill antibacterial system contributed to the treatment of IAIs and the development of innovative antimicrobial strategies.

## 4 Experimental Section

### Synthesis of fascaplysin derivative 14 (F‐14)

The synthetic approaches of F‐14 were based on published literature, with modifications to the cyclization process.^[^ ^59^, ^60^, ^61^ ^]^ β‐carboline 5 (67 mmol) was placed in a stainless‐steel hydrothermal reactor with 2 mL ethylene glycol under a nitrogen atmosphere and heated to 220–240 °C for 1 h. Afterward, the reaction solutions were diluted with 2 mL methanol and then introduced into 35 mL ethyl acetate to precipitate the product. This procedure was repeated until the precipitated product reached purity. β‐carboline 5 was synthesized following previously reported methods.^[^ ^15^ ^]^ The structure of F‐14 was characterized via H nuclear magnetic resonance (^1^H NMR, Bruker 400 MHz, Germany) spectroscopy.

### Fabrication of TiO_2_ Nanostructures (TNA)

Under our previous report,^[^ ^43^ ^]^TNA were prepared on the surface of titanium (Ti) sheets (10 mm × 1 mm, 99.5% purity) and Ti rods (15 mm × 2 mm, 99.5% purity) at a constant voltage of 20 V for 40 min via an anodization process.

### Fabrication of F‐14‐Loaded TNAs

F‐14‐loaded TNAs (F‐14‐TNAs) and DMSO‐loaded TNAs (D‐TNAs) were prepared via lyophilization and vacuum drying.^[^ ^62^ ^]^ In brief, 6.25 µg mL^−1^ F‐14 dimethyl sulfoxide (DMSO, Aladdin, China) solution. TNA surfaces were cleaned with deionized water before F‐14 was doped. 5 µL F‐14 DMSO solution was pipetted onto the TNA surface and gently spread to ensure even coverage. The surfaces were then dried under vacuum at room temperature for 2 h. This loading process was repeated until the nanotube array contained the desired quantity of F‐14 or DMSO.

### Characterization of Material

The structure of each sample was characterized via Fourier transform infrared spectroscopy (FTIR, PerkinElmer PHI 5400, USA) and X‐ray photoelectron spectroscopy (XPS, PerkinElmer PHI 5400, USA). Field emission scanning electron microscopy (FE‐SEM, Hitachi SU‐70, Japan) and energy dispersive spectroscopy (EDS, Inca X‐Max 50, UK) were utilized to observe the micromorphology and elemental composition of the samples. The samples used in the tensile tests were prepared according to ASTM E8‐04 and ASTM E9‐89 standards, and universal material testing equipment (Instron 5969, USA) was used to determine the mechanical properties of the materials at room temperature. In the tensile test, the strain rate was set at 1 × 10^−4^ s^−1^. The yield strength (YS) was defined as the stress leading to 0.2% plastic deformation. Water contact angles were measured via a contact angle instrument (Kruss DSA25, Germany). Transmission electron microscopy (TEM, Thermo Fisher Scientific‐Talos F200S, USA) and energy dispersive spectroscopy (Oxford X‐MaxN 80T IE250, USA) were utilized to observe the images and elemental mapping of MRSA. Scratch test (Rtec SMT‐5000, USA) to assess the mechanical strength of the functional layer on implant surfaces.

### Release from TNA

Ultraviolet (UV) absorption spectroscopy was used to examine F‐14 released from the TNA at various time intervals. The F‐14‐loaded TNAs were immersed in pH 7.4 and pH 5.5 buffer solutions at 37 °C. The release of F‐14 from TNA was measured at various times. The UV absorption peaks of TNA at various concentrations (0.25, 0.5, 1, 2, and 4 µg mL^−1^) at a wavelength of 263 nm were measured to obtain the standard curve. According to the standard curve, the amount of F‐14 released from the TNA was calculated from the absorbance profile.

### Bacterial Virulence Selective Evaluation of TNA In Vitro

Diluted GFP‐labeled *Staphylococcus aureus* (*S. aureus*, ATCC 25 937) and *Staphylococcus epidermidis bacteria* (*S. epidermidis*, ATCC 35 984) solution (10^6^ CFU mL^−1^) were added to the TNA samples and co‐cultured for 24 h. The specimen underwent gentle washing with PBS buffer three times, and bacteria were incubated with 4′,6‐diamidino‐2‐phenylindole (DAPI) to stain nuclei. Subsequently, bacteria were visualized by confocal laser scanning microscopy (CLSM).

In the bacterial agglutination and trap assay, MRSA, *S. aureus*, and *S. epidermidis* were co‐cultured with Ti, TNA, D‐TNA, and F‐14‐TNA, respectively, for 24 h.

### In Vitro Antibacterial Property Assay

Methicillin‐resistant *Staphylococcus aureus* (MRSA, ATCC 43 300) was used for the in vitro antibacterial property assay.

### Antibacterial Property Assay of Implant Surfaces

Four groups of test samples, Ti, TNA, D‐TNA, and F‐14‐TNA, were cocultured with bacteria in the logarithmic growth phase (10^6^ CFU mL^−1^) for 24 h. The specimen underwent gentle washing with PBS buffer three times, and the material samples were subsequently collected.

To detach the bacteria from the material's surface, the tube underwent sonication using an ultrasonic water washing instrument (KQ‐600, China) for 5 min. The sonicated bacterial suspension was serially diluted and subsequently inoculated onto PCA plates. After 24 h of incubation, bacterial colonies were counted.

To observe the quantitative and morphological changes of bacterial adhesion on the surface of the samples, MRSA were fixed with 2.5% glutaraldehyde (Aladdin, China) at 4 °C for 4 h, washed three times with PBS, dehydrated by gradient with different concentrations of ethanol (25%, 50%, 75%, 95%, 100%, 100%) for 10 mins each, and finally allowed to air dry overnight and observed by SEM.

To observe leakage of MRSA cellular contents, the dehydrated samples were embedded in epoxy resin, prepared thin sections using an ultramicrotome, and observed them using a TEM.

### Eradicated Planktobacteria Property Assay of F‐14‐TNA

Four groups of test samples, Ti, TNA, D‐TNA, and F‐14‐TNA, were cocultured with MRSA in the logarithmic growth phase (10^6^ CFU mL^−1^) for 24 h. The bacterial suspension was collected. The eradicated planktobacteria properties were evaluated via the dilution plate counting method and CLSM bacterial Live/Dead staining (Thermo Fisher Scientific, USA).

### Anti‐Biofilm Formation Property Assay

Four groups of test samples, Ti, TNA, D‐TNA, and F‐14‐TNA, were cocultured with bacteria in the logarithmic growth phase (10^6^ CFU mL^−1^) for 24 h. After 24 h, anti‐biofilm formation on the surface of the test samples was evaluated via crystal violet staining (Solarbio, China) and further analyzed via CLSM Live/Dead staining (Thermo Fisher Scientific, USA).

### Lipoteichoic A Competition Assay

The binding affinity of LTA was assessed via a competitive assay, employing the fluorescent probe BODIPY‐TR‐cadaverine (MCE, China). This probe demonstrates fluorescence dequenching upon target engagement. Ti, TNA, D‐TNA, and F‐14‐TNA were cocultured with bacteria in the logarithmic growth phase (10^6^ CFU mL^−1^) for 24 h. The relative intensity of fluorescence was measured at the excitation wavelength of 588 nm and emission wavelength of 616 nm, using the Infinite M200 Pro multifunctional enzyme marker (Tecan, Austria).

### Cytocompatibility Assay of F‐14‐TNA

Rat bone mesenchymal stem cells (BMSCs) were extracted according to our previous method.^[^ ^63^ ^]^ The BMSCs and MC3T3‐E1 were used for the in vitro cytocompatibility assay. Four groups of test samples, Ti, TNA, D‐TNA, and F‐14‐TNA, were cocultured with BMSCs cells (5 × 10^3^ cells well^−1^) for 24 h. The viability of BMSCs cells on the surface of the test samples was evaluated via the CCK‐8 assay. Additionally, the cocultured BMSCs cells and MC3T3‐E1 (2 × 10^4^ cells well^−1^) were subjected to Live/Dead cell staining (Beyotime, China) and cytoskeleton staining (the cytoskeleton was labeled using FITC‐phalloidin, and the nuclei were stained with DAPI, Solarbio, China). The results were then observed using a fluorescence microscope (Olympus, Japan).

### In Vitro Osteogenic Property Assay

BMSCs cells were seeded at a density of 5 × 10^4^ cells per well on various substrates within a 24‐well plate. To facilitate osteogenic differentiation, the culture medium was modified to an osteoinductive medium. This medium consisted of ascorbic acid (50 µg mL^−1^), 10 nM dexamethasone (Aladdin, China), and 10 mM β‐glycerophosphate (Sigma‐Aldrich, USA). The culture medium was refreshed every 2 days. Alkaline phosphatase (ALP) activity was assessed on day 7 utilizing a BCIP/NBT ALP colorimetric assay kit (Beyotime, China). Alizarin red staining (ARS) was assessed on day 21 utilizing 1% ARS solution (pH 4.2; Solarbio, China). Quantitative analysis was performed using imageJ. For the western blotting analysis of XB130, Src, PI3K, and Akt, BMSCs cells were incubated with specific primary antibodies: rabbit anti‐XB130 (1:2000, Affinity, Australia), rabbit anti‐p‐Src (1:1000, Cell Signaling Technology, USA), rabbit anti‐Src (1:1000, Cell Signaling Technology, USA), rabbit anti‐p‐PI3K (1:1000, Cell Signaling Technology, USA), rabbit anti‐PI3K (1:1000, Cell Signaling Technology, USA), rabbit anti‐p‐Akt (1:1000, Cell Signaling Technology, USA), rabbit anti‐Akt (1:1000, Cell Signaling Technology, USA), and rabbit anti‐GAPDH (1:10 000, ABclonal, China). The secondary antibody used was a HRP goat anti‐rabbit Goat Anti‐Rabbit IgG(H+L) antibody (1:50 000, Elabscience, China). The membranes were incubated with enhanced chemiluminescence reagents (NCM, China). For the immunofluorescence analysis of OCN, OPN, and RUNX2, BMSCs cells were incubated with specific primary antibodies: rabbit anti‐OCN (1:400, Boster, China), rabbit anti‐OPN (1:400, Proteintech, China), and rabbit anti‐RUNX2 (1:400, Proteintech, China). The secondary antibody used was a goat anti‐rabbit Alexa Fluor 488 antibody (1:500, Proteintech, China). Cells were counterstained for the cytoskeleton with fluorescein TRITC–labeled phalloidin (Solarbio, China) and nuclei with DAPI. The results were then observed using fluorescence microscope (Olympus, Japan).

### In Vitro Inhibition of Osteoclastic Differentiation

Bone marrow‐derived macrophages were obtained from 6‐week‐old Sprague Dawley (SD) rats and seeded in 24 Wells at equal cell densities. The cells were first cultured in α‐MEM complete medium with 40 ng mL^−1^ M‐CSF for 3 days and then in α‐MEM complete medium with 20 ng mL^−1^ RANKL (Amizona, China) and 40 ng mL^−1^ M‐CSF (Amizona, China) for 6 days, and the solution was changed every 3 days. Fluorescence staining of F‐actin rings was performed when the osteoclasts reached 80–90% density. For F‐actin ring fluorescence visualization, cells were incubated with TRITC‐phalloidin to label F‐actin and 4′,6‐diamidino‐2‐phenylindole (DAPI) to counterstain nuclei.

### In Vitro Macrophage Activation

RAW 264.7 cells were treated with lipopolysaccharide (Sigma‐Aldrich, USA) to induce macrophage polarization and subsequently cultured with various treatment groups for 24 h. Flow cytometry and immunofluorescence staining was performed to assess CD86 and CD206 expression. Primary antibodies utilized were rabbit anti‐CD206 (1:400, Proteintech, China) and rat anti‐CD86 (1:400, Proteintech, China). Secondary antibodies included goat anti‐rabbit Alexa Fluor 488 (1:400, Proteintech, China) and goat anti‐rat Alexa Fluor 594 (1:400, Invitrogen, USA). Following staining, nuclei were counterstained with DAPI. Fluorescence intensity and imaging were conducted using the CLSM. Gene expression of pro‐inflammatory cytokines (IL‐1β) and anti‐inflammatory cytokines (IL‐10) was quantified via quantitative reverse transcription polymerase chain reaction. GAPDH served as the housekeeping gene, and primer sequences are detailed in Table S1, Supporting Information.

### mRNA Transcriptomes Sequencing

To investigate the antibacterial mechanism of F‐14‐TNA against MRSA, four various treatments, Ti, TNA, D‐TNA, and F‐14‐TNA, were cocultured with MRSA bacteria in the logarithmic growth phase (10^10^ CFU mL^−1^) for 12 h. After that, total bacterial RNA was extracted, and RNA sequencing was performed. A whole‐genome sequencing workflow (Illumina Novase 6000, USA) was used for data analysis. DESeq2 (Illumina Novase 6000, USA) was used to compare gene expression differences among the F‐14‐TNA, Ti, TNA, and D‐TNA groups.

### Real‐Time Quantitative Polymerase Chain Reaction Analysis

Four treatment groups, Ti, TNA, D‐TNA, and F‐14‐TNA, were cocultured with MRSA bacteria in the logarithmic phase (10^10^ CFU mL^−1^) for 12 h. Total bacterial RNA was extracted via lysostaphin (Sigma, USA) and TRIzol reagent (Thermofisher, USA), and the extracted RNA was then transcribed into complementary DNA (cDNA). The primer sequences are detailed in Table S1, Supporting Information. The expression levels of the PTSI, icaA, and icaD genes were quantified through RT‒qPCR, and 16S rRNA was chosen as the reference gene in this study.

### Measurement of Intracellular cAMP Levels

The intracellular cAMP levels of MRSA cocultured with Ti, TNA, D‐TNA, and F‐14‐TNA were measured via a cAMP Elist Kit (NewEast Biosciences, China) following the manufacturer's instructions. Briefly, four treatments, Ti, TNA, D‐TNA, and F‐14‐TNA, were cocultured with MRSA bacteria in the logarithmic growth phase (10^10^ CFU mL^−1^) for 12 h, and the intracellular cAMP levels were determined via a microplate reader (Thermo Fisher, USA).

### Measurement of the Intracellular ATP Level

An enhanced ATP assay kit (Beyotime, China) was used to detect the intracellular ATP levels of MRSA treated with Ti, TNA, D‐TNA, or F‐14‐TNA following the manufacturer's instructions. In brief, various treatments, namely, Ti, TNA, D‐TNA, and F‐14‐TNA, were cocultured with MRSA bacteria in the logarithmic growth phase (10^10^ CFU mL^−1^) for 12 h, and the intracellular ATP levels were determined via the Infinite M200 Pro multifunctional enzyme marker (Tecan, Austria).

### ROS Measurement

The production of ROS in MRSA cocultured with Ti, TNA, D‐TNA, and F‐14‐TNA was quantified via a fluorescent probe (2′,7′‐dichlorodihydrofluorescein diacetate (DCFH‐DA)) following the manufacturer's instructions (Beyotime, China). In brief, various treatments, Ti, TNA, D‐TNA, and F‐14‐TNA, were cocultured with MRSA bacteria in the logarithmic phase (10^10^ CFU mL^−1^) for 12 h. After 12 h, the level of ROS production was determined via flow cytometry (FCM, Beckman Coulter, United States) and CLSM.

### In Vivo Antibacterial Evaluation of F‐14‐TNAs Against MRSA–Intramedullary Implant Infection Model

All animal surgical procedures were approved by the Animal Ethics Committee of Ningbo University with approval number NBU20220237. Male Sprague–Dawley (SD) rats (8 weeks old) were divided into five groups according to different processing methods. The SD rats in this study were anesthetized via subcutaneous injection of sodium pentobarbital (80 mg kg^−1^). A hole was created in the distal femur in the direction of the medullary cavity with a drill bit of φ2.5 mm. The noninfected group (control group) underwent drilling without the implantation of metal rods. For the infected group, metallic rods (TNA, D‐TNA, and F‐14‐TNA) were immersed in 1 mL of MRSA suspension at a concentration of 1 × 10^6^ CFU mL^−1^ and cultured at 37 °C for 24 h to form MRSA biofilms.^[^ ^24^ ^]^ After implantation, the soft tissue and skin were sutured separately. In addition, all SD rats were fed a standard diet. Tissue samples were collected at 2 and 4 weeks after implantation for subsequent analysis. Organ and blood samples were collected at 4 weeks after implantation for subsequent analysis.

### General Condition of the Animals and Evaluation of Hematological Parameters

After the operation, the body weights and body temperatures of all the experimental animals were monitored regularly.

### In Vivo Microbiological Evaluation

At 2 and 4 weeks after implantation, all the SD rats were euthanized to obtain the right femur, and the metal rods were carefully removed from the femur specimens. Three femur samples were then randomly chosen and subjected to ultrasonic treatment to collect bacteria adhering to both the implant surface and the bone tissue. The bacterial suspension was subsequently diluted, plated, cultured, and counted. The bacterial colony‐forming units (CFUs) per plate were then enumerated to evaluate the number of bacteria adhering to the implant surface and bone tissue.

### Micro‐Computed Tomography

After being immersed in 4% tissue cell fixative (Solarbio, China), the rat femur samples were scanned via micro‐CT (PINGSENG Healthcare Inc., China). After scanning and reconstruction, the cylindrical region with a height of 1 millimeter and a circumference of 1 millimeter away from the periphery of the femoral medullary cavity metal rod (excluding intramedullary nails) was defined as the region of interest (ROI). In this region, the bone mineral density (BMD), bone volume (BV), bone volume fraction (BV/TV), trabecular number (Tb.N), trabecular thickness (Tb.Th), and trabecular separation (Tb.Sp) were calculated.

### Histological Analysis

The femoral samples were fixed with 4% paraformaldehyde, then 10% EDTA decalcified, and finally, the metal rods were carefully removed from the femoral samples. All the rat femur samples were dehydrated with graded alcohol (70%, 80%, 90%, 95%, 100%, and 100%) and xylene, and the femur samples were embedded in paraffin and sectioned for H&E, Gram, Masson, toluidine blue (TB), TRAP staining, and then observed by microscopy. IF was conducted using anti‐CD206 (Abcam, USA), CD68 Ab (Proteintech, China), Runx2 Ab (BOSTER, China), and FITC/Cy3 labelled Goat Anti‐Rabbit IgG (Proteintech, China). IF was observed by fluorescence microscopy. Immunohistochemistry (IHC) was conducted using anti‐OCN (BOSTER, China) and Goat Anti‐Rabbit IgG (Elabscience, China). IHC was observed by microscopy.

### Statistical Analysis

GraphPad Prism 9 was used for data analysis. The sample size (n) and statistical methods used to test significant differences were indicated in the figure legends. The quantitative data are expressed as the mean ± standard deviation (SD). The data were analyzed via an independent sample *t‐*test and one‐way analysis of variance (ANOVA). The *p* value was set at *p < 0.05, **p < 0.01, or ***p < 0.001 to indicate statistically significant differences.

## Conflict of Interest

The authors declare no conflict of interest.

## Author Contributions

X.H. and J.Z. contributed equally to this work. X.H.: Writing – original draft, Methodology, Investigation, Data curation, Conceptualization. J.Z.: Methodology, Investigation, Data curation, Conceptualization. Y. C.: Methodology, Investigation, Data curation. B.L.: Methodology, Investigation. T. D.: Methodology, Investigation, Data curation. W.Z.: Investigation, Software. M.Z.: Methodology, Investigation. H.L.: Supervision, Resources. J.N.: Writing – review & editing, Supervision, Conceptualization. Z.P.: Writing – review & editing, Supervision, Project administration, Funding acquisition, Conceptualization. All authors read and approved the final manuscript.

## Supporting information



Supporting Information

## Data Availability

The data that support the findings of this study are available from the corresponding author upon reasonable request.;
