# Supplementary material for: Virulence‐Selective Trap–Capture–Kill Antibacterial Nanostructures With Immune–Metabolic Regulation for Treating Implant‐Associated Infections
Source: Adv Sci (Weinh). 2025 Sep 15;12(44):e11045. doi: 10.1002/advs.202511045 (PMC12667538; doi:10.1002/advs.202511045)
Supplement: Supplementary file 1 — Supporting Information [file ADVS-12-e11045-s001.docx]

**Virulence-Selective Trap-Capture-Kill Antibacterial Nanostructures with Immune-Metabolic Regulation for Treating Implant-Associated Infections**

Xiaodong Hu^1,2,^, Jiaqi Zhong^1,2,^, Yujiong Chen^1^, Botao Liu^1^, Tianyu Du^2^, Weilai Zhu^3^, Minzhe Zheng^1^, Hongze Liang^4,*^, Jiahua Ni^5,*^, Zhaoxiang Peng^1,2,*^

^1^ Affiliated LiHuiLi Hospital, Ningbo University, Ningbo 315040, China

^2^ Health Science Center, Ningbo University, Ningbo 315211, China

^3^ Peking University First Hospital, Beijing 100034, China

^4^ Key Laboratory of Advanced Mass Spectrometry and Molecular Analysis of Zhejiang Province, School of Materials Science and Chemical Engineering, Ningbo University, Ningbo, 315211, China

^5^ College of Biological Science and Medical Engineering, Donghua University, Shanghai 201620, China

* Corresponding authors.

E-mail addresses: [lianghongze@nbu.edu.cn](mailto:lianghongze@nbu.edu.cn) (H. Liang). jiahua.ni@dhu.edu.cn (J. Ni), lhlpengzhaoxiang@nbu.edu.cn (Z. Peng).

X.H. and J.Z. contributed equally to this work.

Supplementary materials

Table S1. The primer sequence used for RT-qPCR analysis

| IL10 | Forward | ACTACCAAAGCCACAAGGCA |
| --- | --- | --- |
|  | Reverse | ACACCTTGGTCTTGGAGCTTATTA |
| IL1β | Forward | TGCCACCTTTTGACAGTGATG |
|  | Reverse | TTCTTGTGACCCTGAGCGAC |
| GAPDH | Forward | AACTTTGGCATTGTGGAAGG |
|  | Reverse | ACACATTGGGGGTAGGAACA |
| PTSI | Forward | TGCGCTAACATTGGTACGGT |
|  | Reverse | ATTTACCGAAAGCCGAGGCA |
| icaA | Forward | CTTGCTGGCGCAGTCAATAC |
|  | Reverse | GTAGCCAACGTCGACAACTG |
| icaD | Forward | TGGGCATTTTCGCGATTATCA |
|  | Reverse | ACGATTCTCTTCCTTTCTGCCA |
| 16S rRNA | Forward | CGCAATGGGCGAAAGC |
|  | Reverse | TACGATCCGAAGACCTTCATCA |

Table S2. Immunocytologic findings in rats at 4 weeks postoperatively.

|  | Control | F-14-TNA |
| --- | --- | --- |
| NEUT% | 21.94±1.94% | 21.77±2.51% |
| LYMP% | 46.02±3.89% | 46.15±3.80% |
| MONO% | 3.80±0.48% | 3.83±3.88% |
| NEUT# | 1.13±0.18×10^9^/L | 1.08±0.15×10^9^/L |
| LYMP# | 2.12±0.19×10^9^/L | 2.08±0.21×10^9^/L |
| MONO# | 0.23±0.1×10^9^/L | 0.23±0.08×10^9^/L |

Data represent the mean ± SD; n=6.

NEUT%, the percentage of neutrophils; LYMP%, the percentage of lymphocytes; MONO%, the percentage of Monocytes; NEUT#, absolute neutrophils; LYMP#, absolute lymphocytes; MONO#, absolute Monocytes.


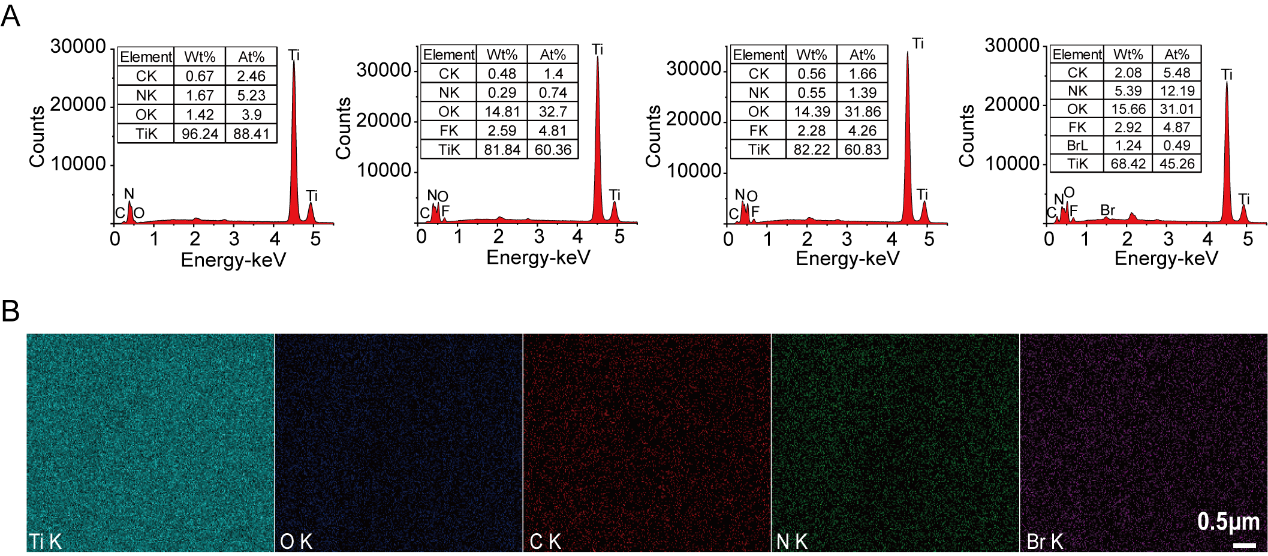


Figure S1. EDS elemental mappings of F-14-TNA.


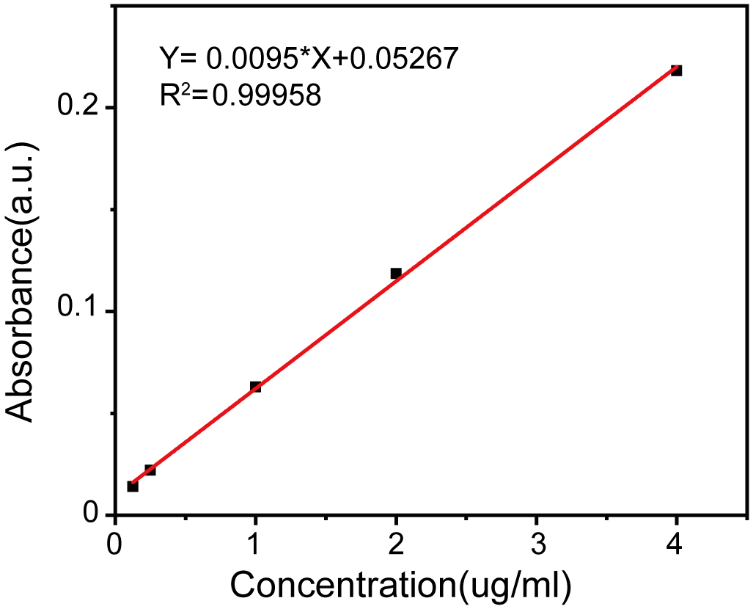


Figure S2. Standard curve


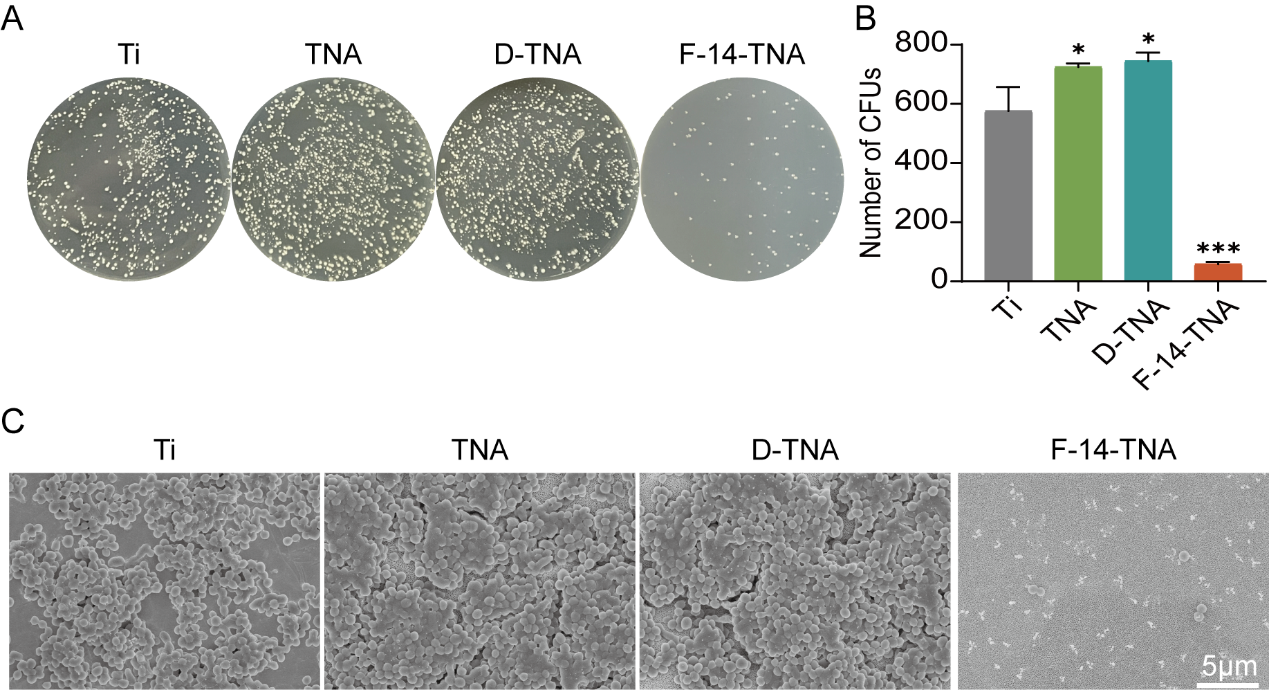


Figure S3. *In vitro* antibacterial evaluation of F-14-TNA. A, B) Images of bacterial cultures derived from implant surfaces were acquired using sonication, and the corresponding number of bacteria was determined. C) SEM photographs of adsorption between Ti, TNA, D-TNA, and F-14-TNA with MRSA. The data represent the mean ± SD; n=3; * p<0.05, *** p < 0.001.


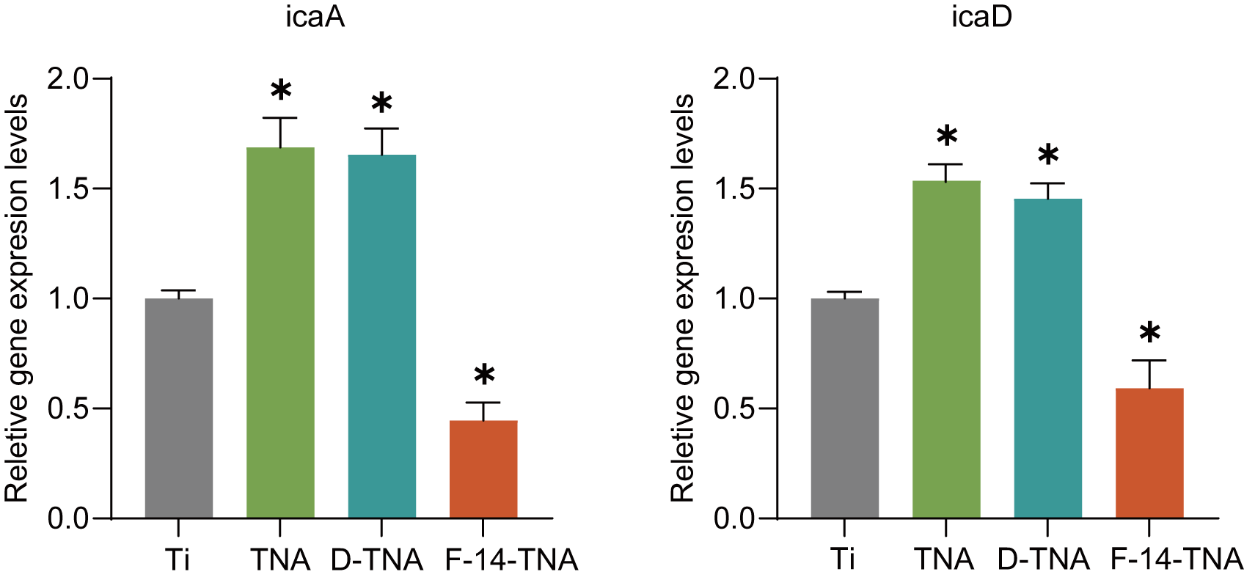


Figure S4. mRNA expression levels of the adhesion gene (icaA and icaD) in MRSA cultured on various samples. The data represent the mean ± SD; n=3; * p<0.05.


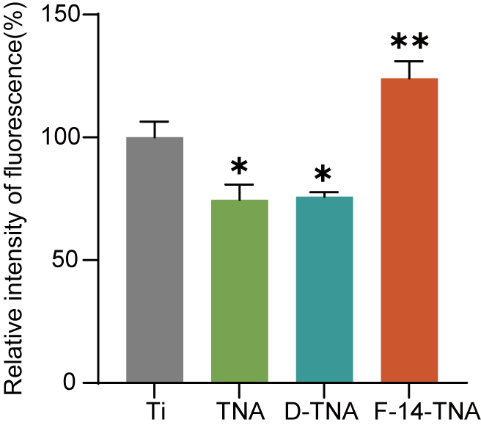


Figure S5. Competitive binding affinity assay between F-14 and the fluorescent probe BODIPY-TR-cadaverine to the LTA. The data represent the mean ± SD; n=3; * p<0.05, ** p < 0.01.


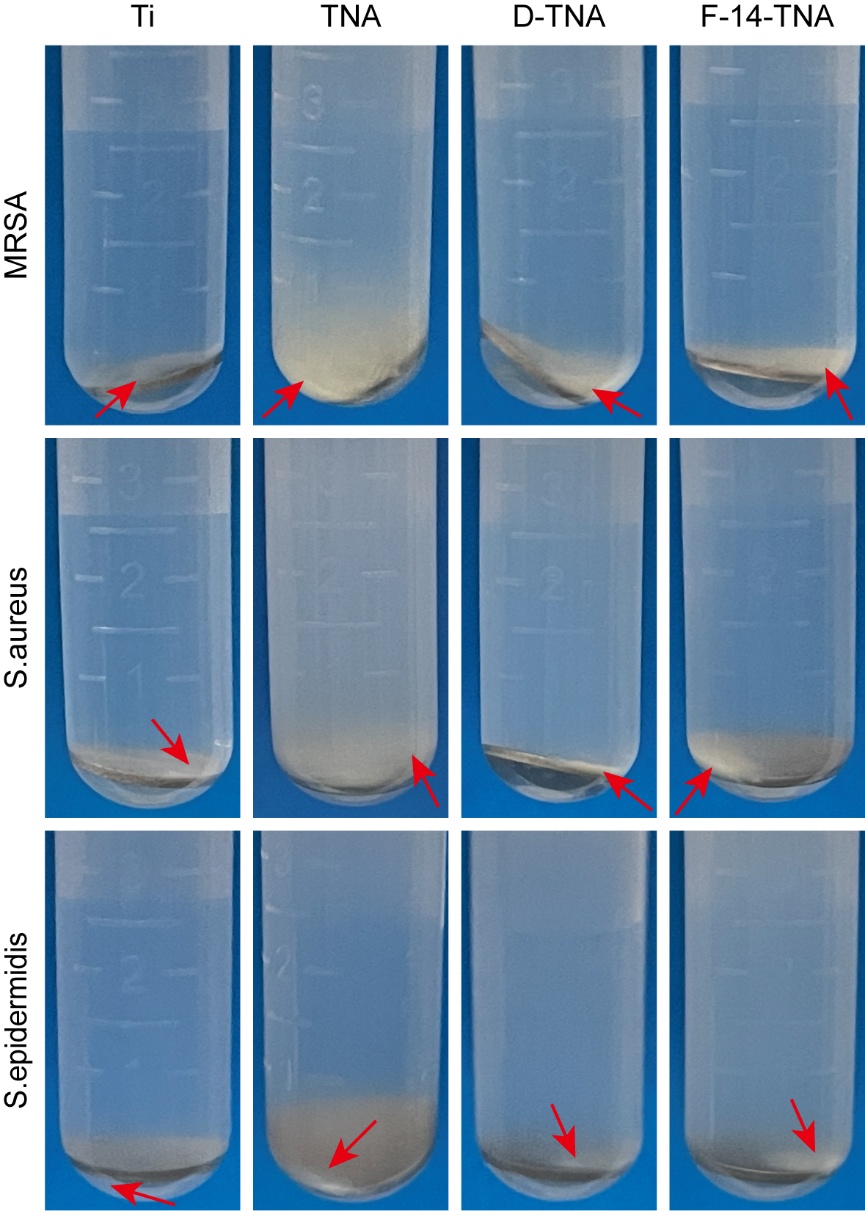


Figure S6. Agglutination and trapping of MRSA, *S. aureus*, and *S. epidermidis* treated with different groups for 12h.


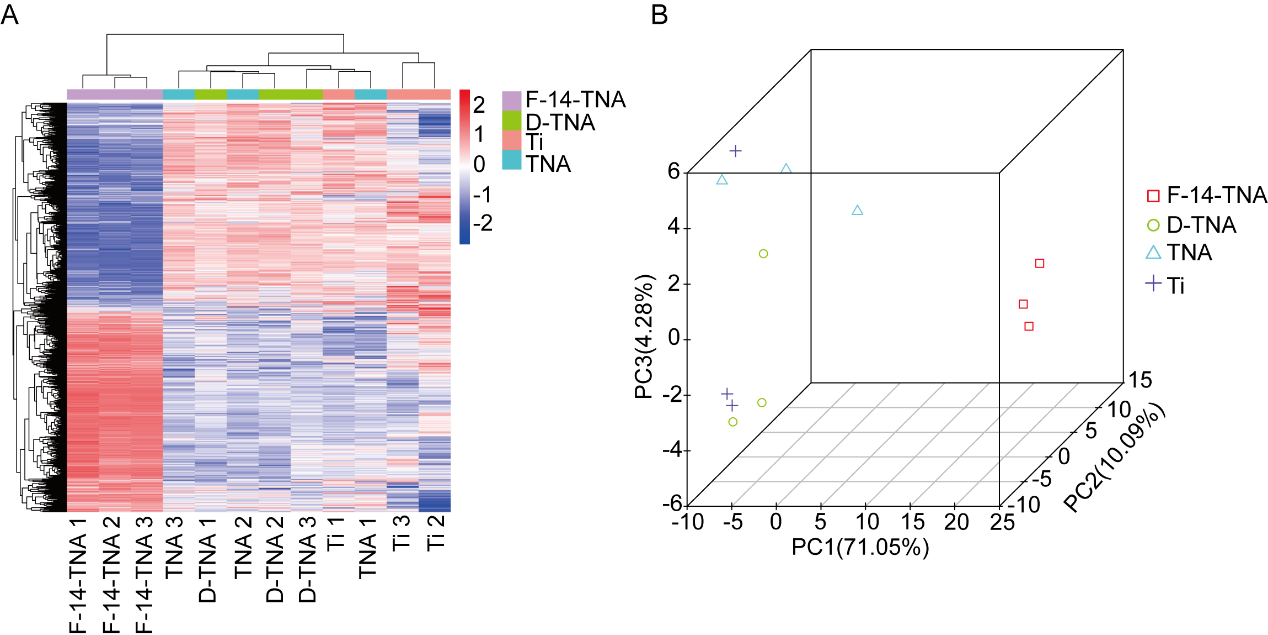


Figure S7. A) Heat map of gene expression of MRSA treated with Ti, TNA, D-TNA, and F-14-TNA groups. B) PCA analysis of gene expression of MRSA treated with Ti, TNA, D-TNA, and F-14-TNA groups.


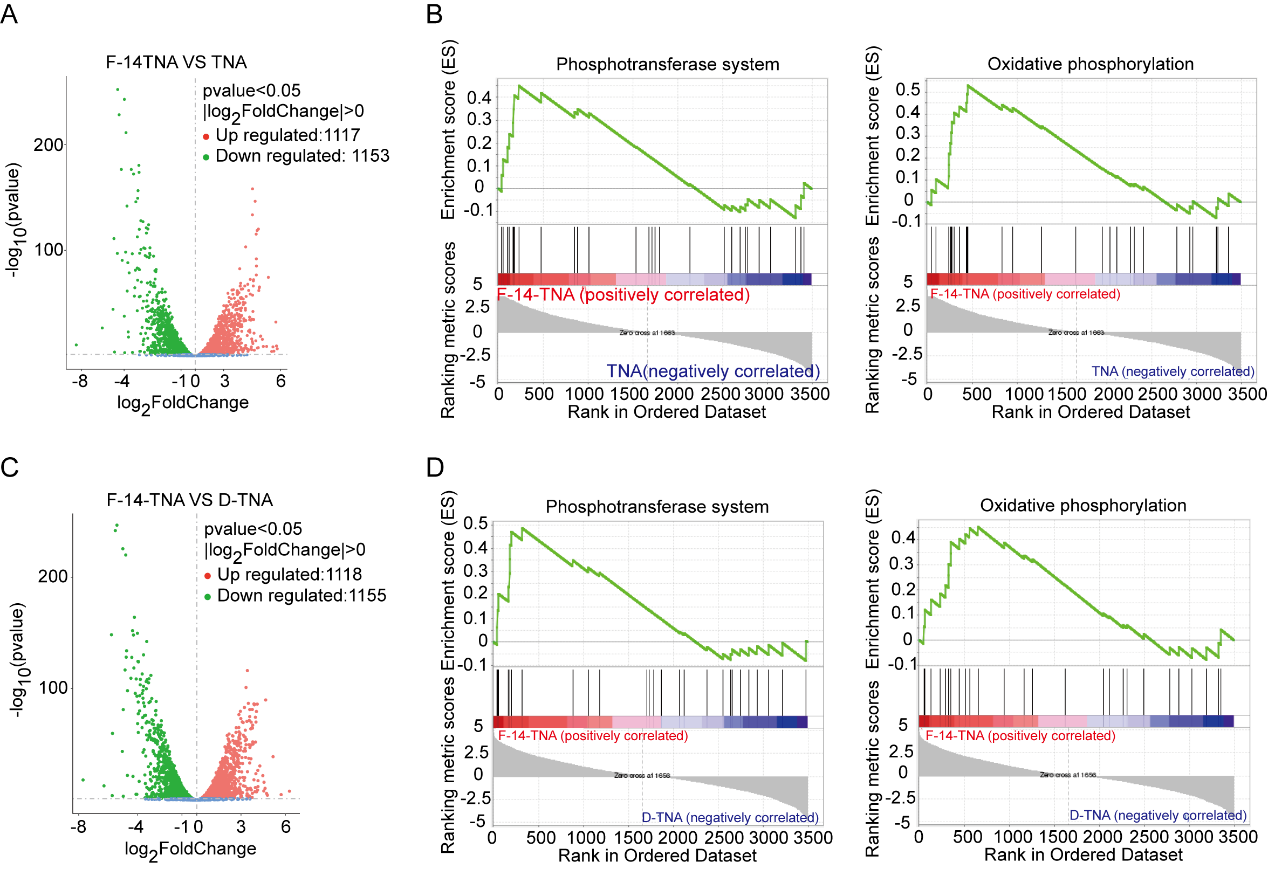


Figure S8. A, C) Volcano plot of differentially expressed genes between TNA, D-TNA, and F-14-TNA treated groups. B, D) The gene set enrichment analysis of the phosphotransferase system and oxidative phosphorylation between TNA, D-TNA, and F-14-TNA treated groups.


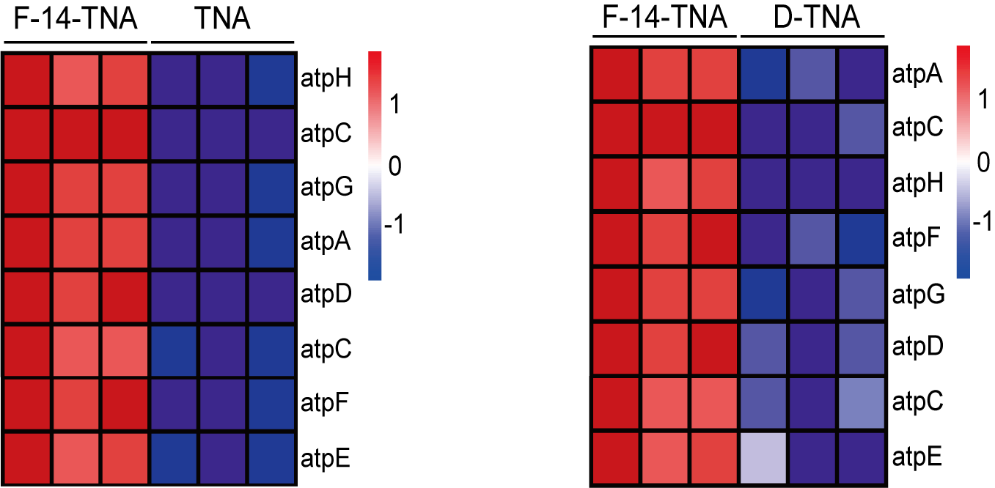


Figure S9. Heat-map of gene expression in bacteria treated with TNA, D-TNA, and F-14-TNA groups.


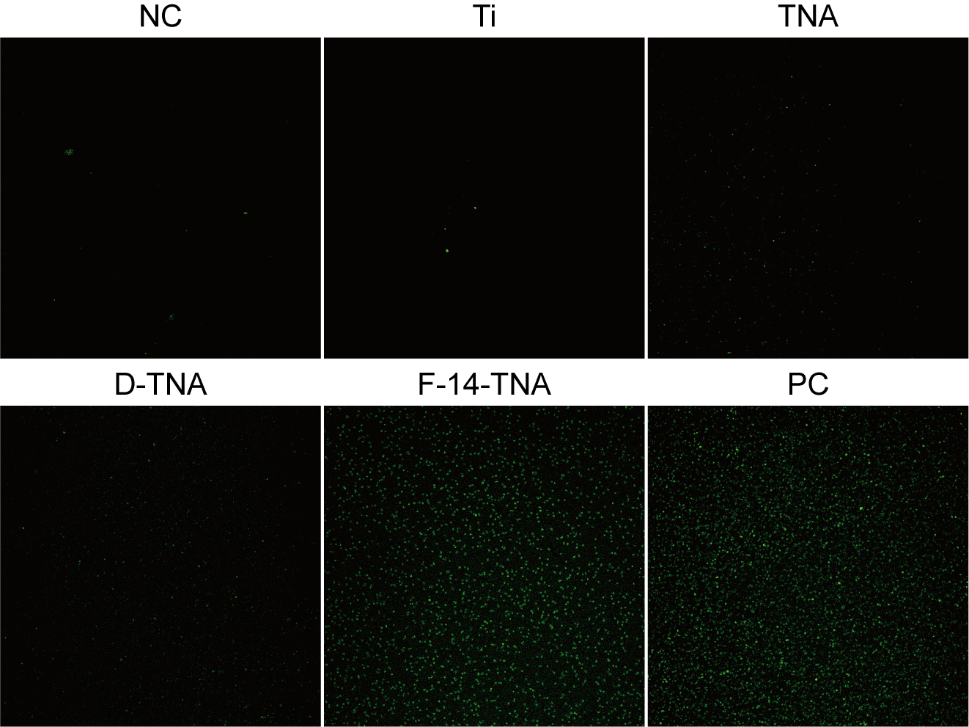


Figure S10. Fluorescence microscopy images of ROS generated by various treatments (NC: negative control group, PC: positive control group).


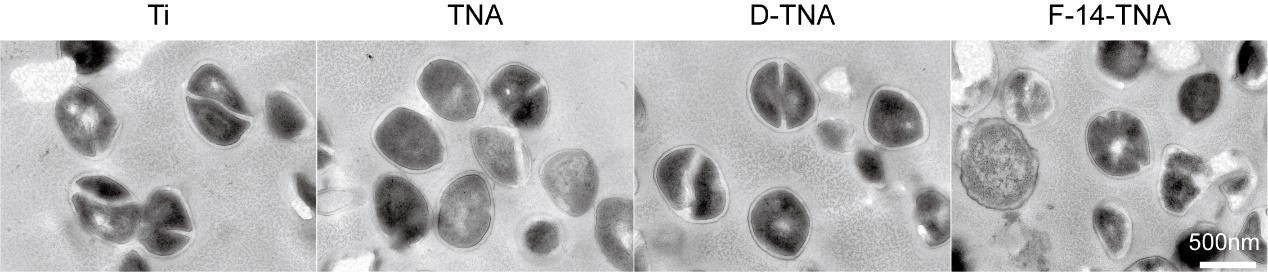


Figure S11. TEM images of MRSA following treatment with various treatments.


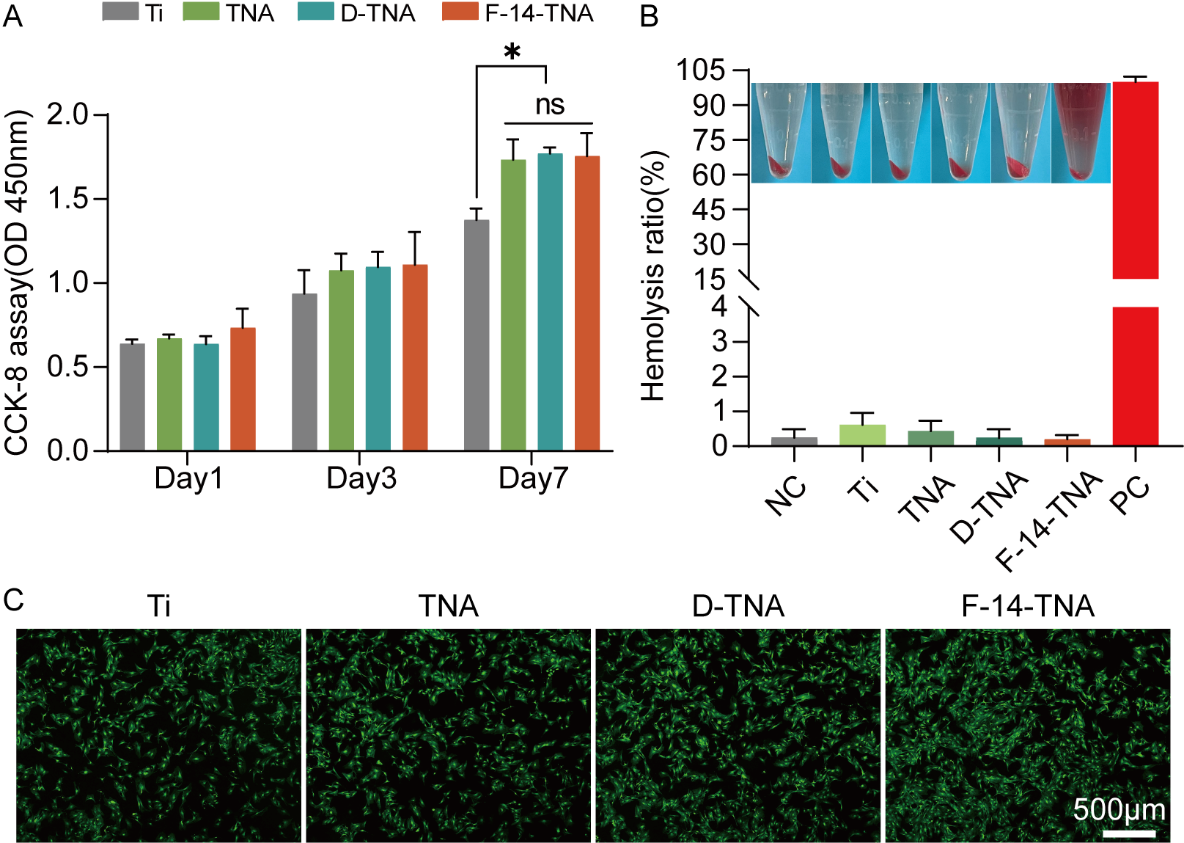


Figure S12. Biocompatibility assessments of F-14-TNA *in vitro*. A) Cell viability assay of BMSCs cultured on Ti, TNA, D-TNA, and F-14-TNA surfaces for 1, 3, and 7 days. B) Blood compatibility assay of Ti, TNA, D-TNA, and F-14-TNA. NC: negative control group, PC: positive control group. C) Live/Dead staining of BMSCs cultured for 1 day on Ti, TNA, D-TNA, and F-14-TNA surfaces. Data represent the mean ± SD; n=6; * p < 0.05.


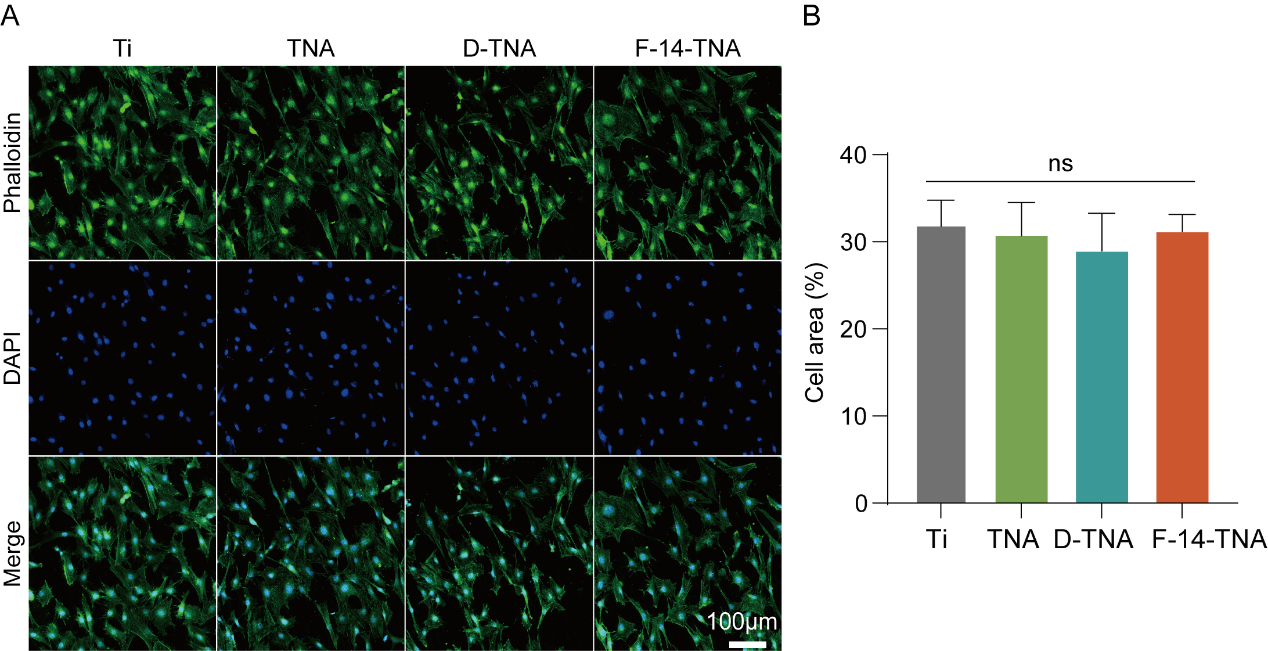


Figure S13. images of cell adhesion derived from implant surfaces (A), and the corresponding cell area was determined (B) (fluorescent green for F-actin, blue for cell nucleus). Data represent the mean ± SD; n=3. ns: not significant.


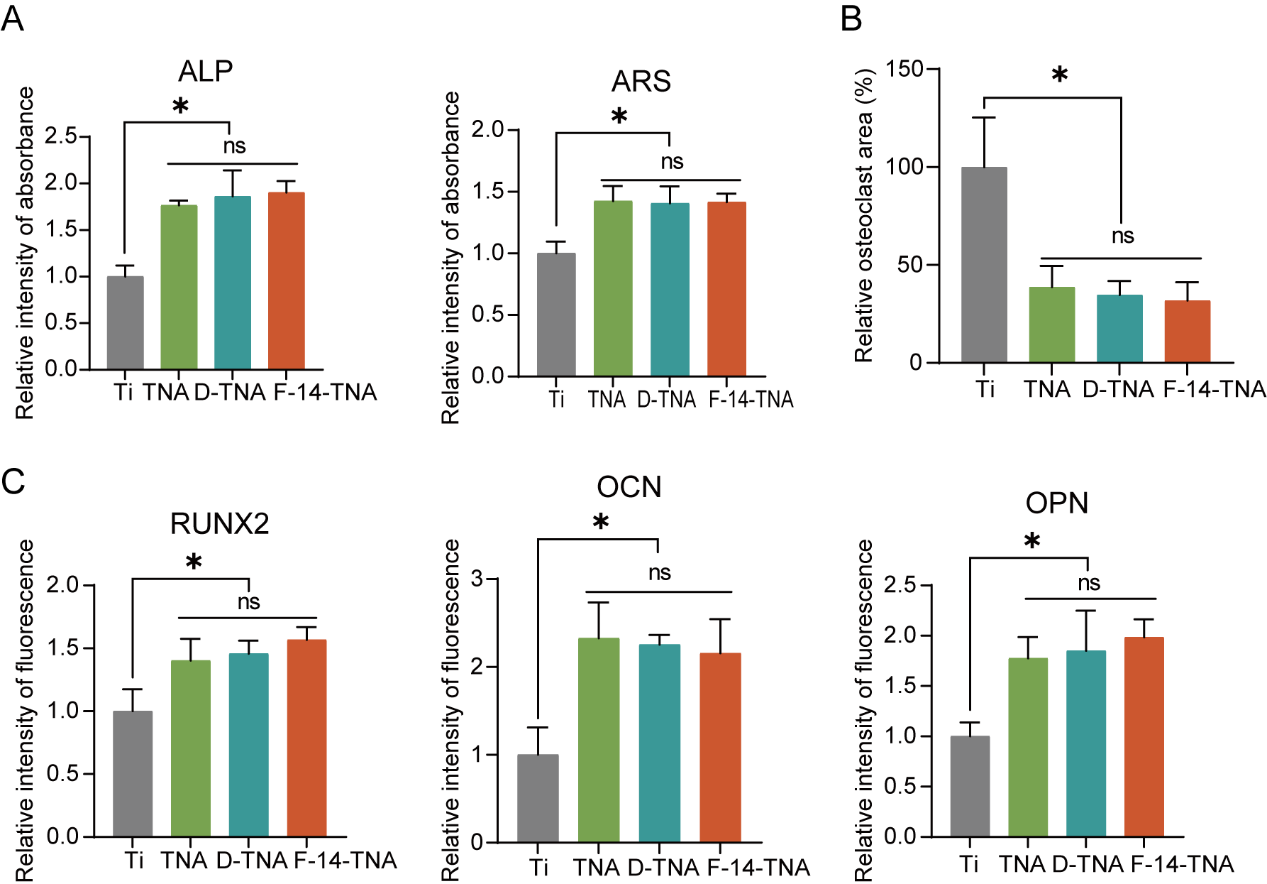


Figure S14. A) Relative quantitative analysis of ALP intensity and ARS intensity. B) Relative quantitative analysis of osteoclast area. C) Relative quantitative analysis of immunoﬂuorescence staining for RUNX2, OCN, and OPN. Data represent the mean ± SD, n=3, *p < 0.05.


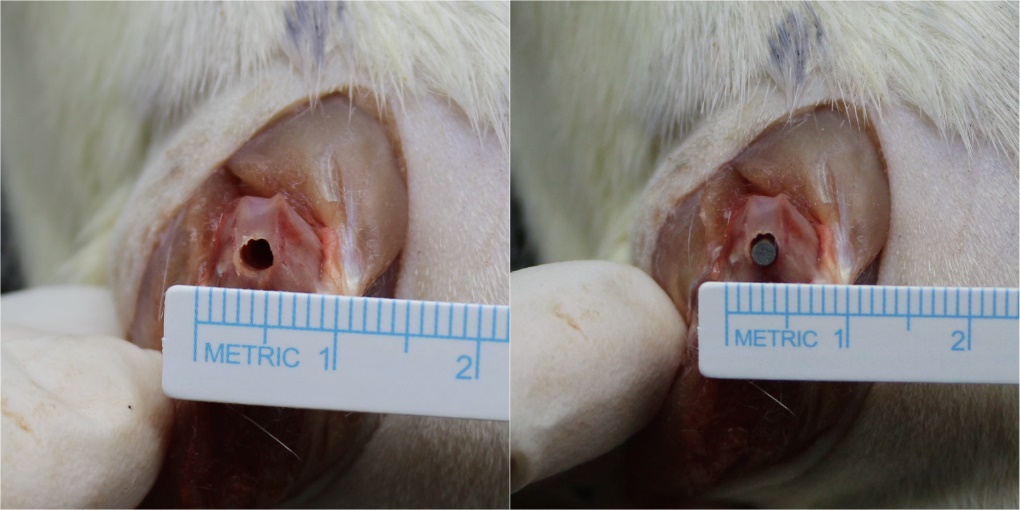


Figure S15. Surgical procedure of MRSA-infected implants implanted into the bone marrow cavity of the femur of SD rats.


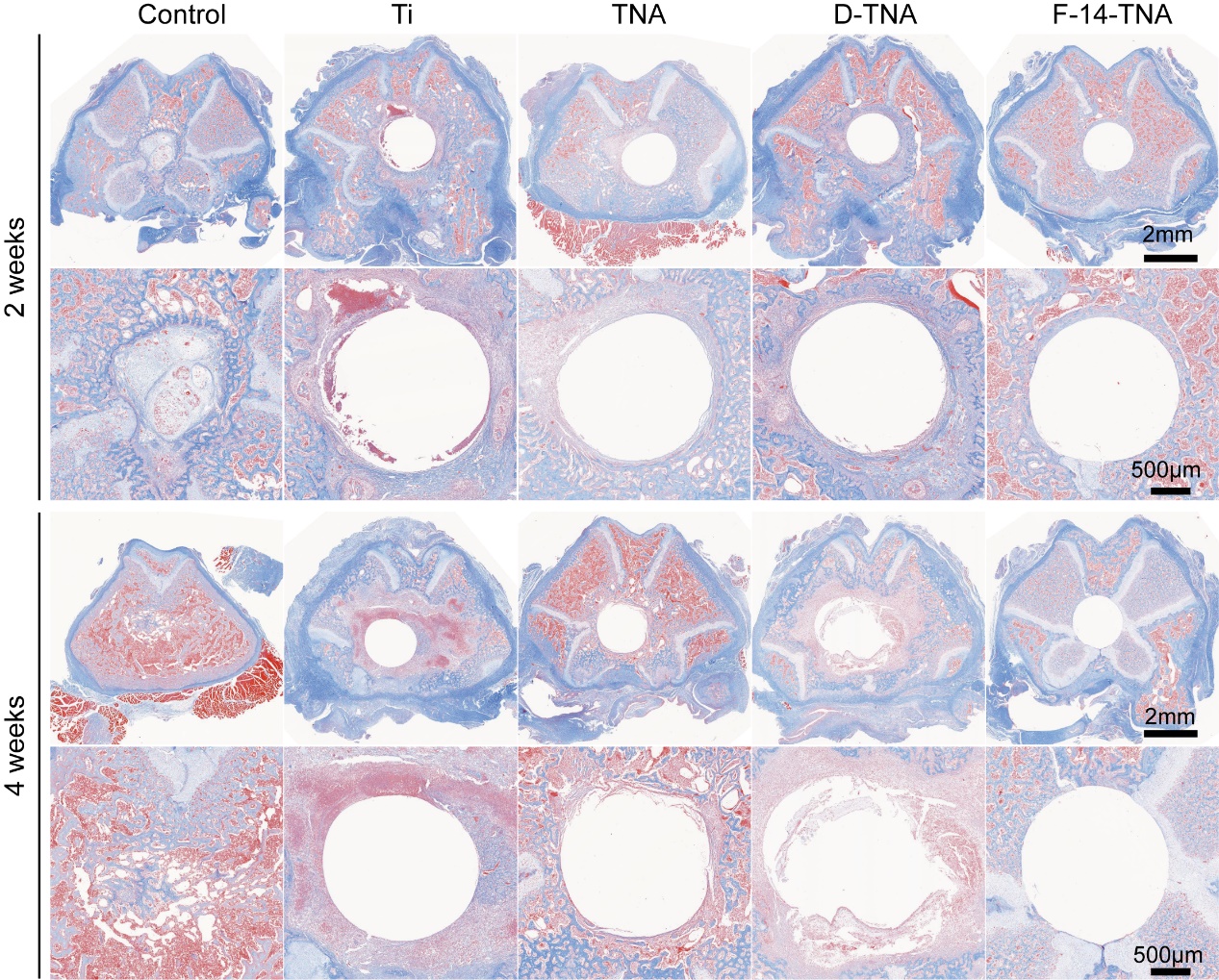


Figure S16. Masson staining of peri-implant bone tissues at 2 and 4 weeks.


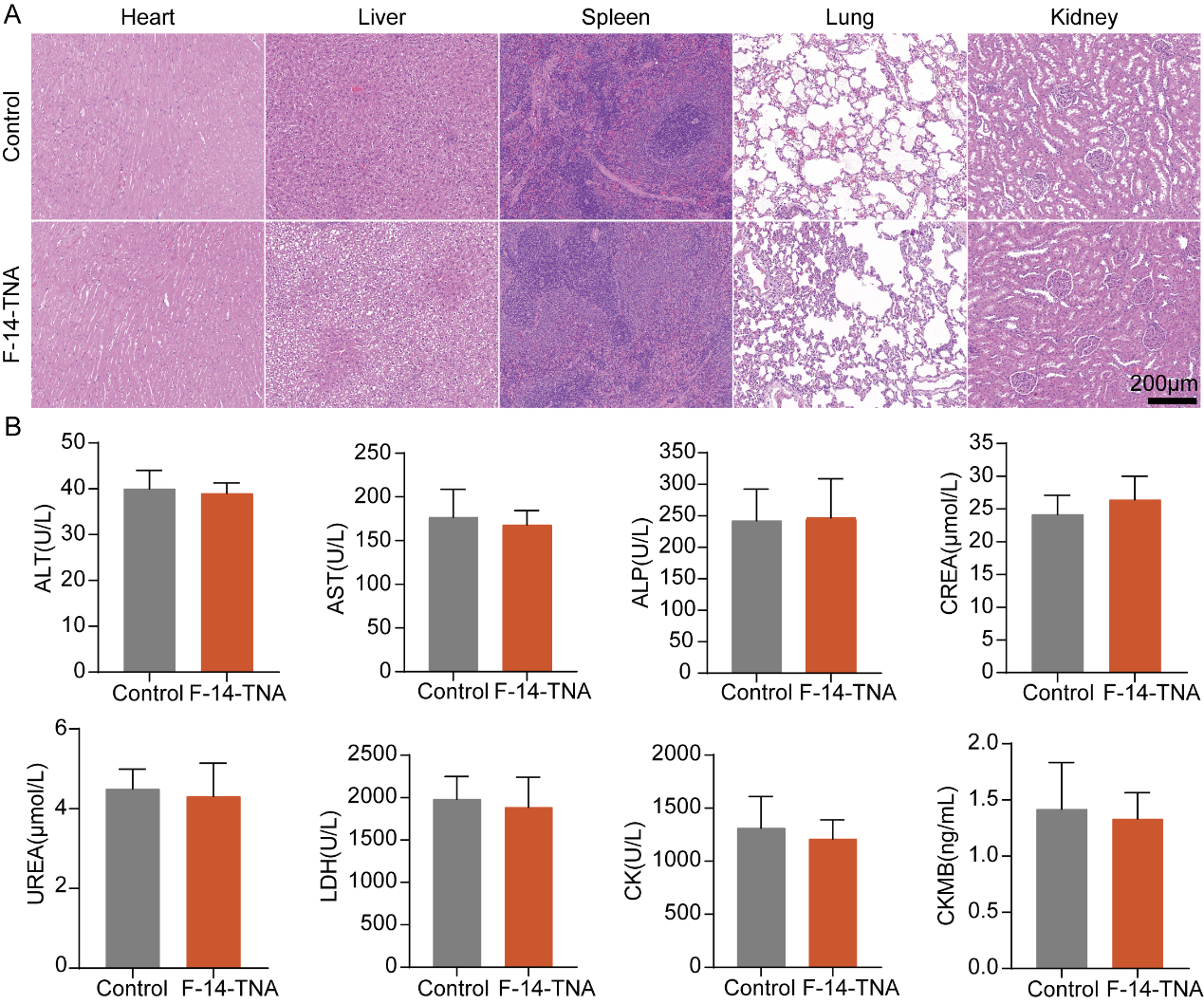


Figure S17. *In vivo* biosafety evaluation of F-14-TNA. A) HE staining results of the heart, liver, spleen, lungs, and kidneys of SD rats in the F-14-TNA group and the control group 4 weeks after surgery. B) Serum biochemical test results of alkaline phosphatase (ALP), glutamic pyruvic transaminase (AST), alkaline phosphatase (ALP), creatinine (CREA), UREA, lactate dehydrogenase (LDH), creatine kinase (CK), and MB isoenzyme of creatine kinase (CKMB). for the F-14-TNA group and the control group 4 weeks after surgery. Data represent the mean ± SD, n=6.
